# A nanofiber-hydrogel composite improves tissue repair in a rat model of Crohn’s disease perianal fistulas

**DOI:** 10.1126/sciadv.ade1067

**Published:** 2023-01-04

**Authors:** Ling Li, Zhi-Cheng Yao, Alyssa Parian, Yueh-Hsun Yang, Jeffrey Chao, Jason Yin, Kevan J. Salimian, Sashank K. Reddy, Atif Zaheer, Susan L. Gearhart, Hai-Quan Mao, Florin M. Selaru

**Affiliations:** ^1^ Division of Gastroenterology and Hepatology, School of Medicine, Johns Hopkins University, Baltimore, MD, USA.; ^2^ Institute for NanoBioTechnology, Johns Hopkins University, Baltimore, MD, USA.; ^3^ Department of Materials Science and Engineering, Whiting School of Engineering, Johns Hopkins University, Baltimore, MD, USA.; ^4^ Translational Tissue Engineering Center, Johns Hopkins University School of Medicine, Baltimore, MD, USA.; ^5^ Department of Plastic and Reconstructive Surgery, Johns Hopkins School of Medicine, Baltimore, MD, USA.; ^6^ Department of Public Health Studies, Krieger School of Arts and Sciences, Johns Hopkins University, Baltimore, MD, USA.; ^7^ Department of Pathology, Johns Hopkins University School of Medicine, Baltimore, MD, USA.; ^8^ Division of Radiology and Radiological Sciences, School of Medicine, Johns Hopkins University, Baltimore, MD, USA.; ^9^ Division of Colorectal Surgery, Department of Surgery, Johns Hopkins University School of Medicine, Baltimore, MD, USA.; ^10^ Department of Biomedical Engineering, Johns Hopkins University School of Medicine, Baltimore, MD, USA.; ^11^ Department of Oncology, Sidney Kimmel Cancer Center, School of Medicine, Johns Hopkins University, Baltimore, MD, USA.

## Abstract

Perianal fistulas (PAFs) represent a severe complication of Crohn’s disease (CD). Despite the advent of biologic and small-molecule therapeutics for luminal disease, PAFs in CD (CD-PAF) are relatively resistant to treatment, with less than 50% responding to any therapy. We report an injectable, biodegradable, mechanically fragmented nanofiber-hydrogel composite (mfNHC) loaded with adipose-derived stem cells (ADSCs) for the treatment of fistulas in a rat model of CD-PAF. The ADSC-loaded mfNHC results in a higher degree of healing when compared to surgical treatment of fistulas, which is a standard treatment. The volume of fistulas treated with mfNHC is decreased sixfold compared to the surgical treatment control. Molecular studies reveal that utilization of mfNHC reduced local inflammation and improved tissue regeneration. This study demonstrates that ADSC-loaded mfNHC is a promising therapy for CD-PAF, and warrants further studies to advance mfNHC toward clinical translation.

## Teaser

An injectable nanofiber-hydrogel composite with adipose-derived stem cells promotes regenerative healing of perianal fistula.

## INTRODUCTION

Inflammatory bowel disease (IBD) includes Crohn’s disease (CD) and ulcerative colitis (UC) and is a complex, multifactorial, immune-mediated illness (*1*, *2*). There are approximately 6.9 million IBD cases globally, and this number is rising (*1*). In 2020, there were approximately 2.5 million IBD cases in the United States, and this number is expected to increase to 3.5 million by 2030 (*2*). Currently, it is estimated that 780,000 patients in the United States suffer from CD, and 33,000 new cases are diagnosed each year (*3*). Perianal fistulas (PAFs) occur in approximately 30 to 40% of CD (*4*–*6*). PAF in CD (CD-PAF) is estimated to affect more than 15,000 new patients each year (*7*). CD-PAF can be associated with perianal pain, purulent or feculent drainage, frank fecal incontinence, and superimposed infections. These complications of CD-PAF are associated with reduced quality of life and morbidity. Current clinical treatment strategies aim to promote long-term fistula healing while avoiding incontinence and diverting stomas (*8*). These approaches, however, are successful in less than 50% of patients (*6*, *9*–*20*). For example, randomized clinical trials showed that infliximab, a tumor necrosis factor–α (TNF-α) antibody that is one of the most effective treatments for luminal inflammation, induces complete closure in a mere 23% of CD-PAF (*18*–*20*). Other medical therapies, including antibiotics or immunomodulators, are even less effective in the long-term treatment of CD-PAF, with as many as 70% of fistulas relapsing upon discontinuation of treatment (*15*–*17*). Another approach in the treatment of CD-PAF is surgical. Unfortunately, surgical interventions to address PAFs in CD are marred by frequent fistula recurrence, as well as local complications such as damage to anal sphincter that can lead to fecal incontinence (*11*, *12*).

A newer treatment of CD-PAF consists of allogeneic adipose-derived stem cells (ADSCs) injected into and around the fistula tract (*6*, *13*, *14*, *21*, *22*). In a phase 3 clinical trial, in addition to surgical treatment of PAFs, a single local administration of 120 million ADSCs into the fistula tract induced clinical PAF remission at week 52 in 59.2% of patients versus 41.6% achieved in the standard surgical treatment with placebo [phosphate-buffered saline (PBS)] injection arm. On the basis of these results, the ADSC treatment has recently been approved by the European Medicines Agency for the treatment of complex CD-PAF that do not respond to conventional and/or biologic therapies (*13*, *14*). This treatment has not yet been approved in the United States. Although it has not been studied specifically for fistulas, it has been hypothesized that the beneficial effects of ADSCs at the site of injury are potentially limited by ADSC migration away from the site (*23*). A potential strategy to improve the efficacy of ADSCs would therefore include a methodology to retain ADSCs within the fistula tract. Another investigational approach to the treatment of PAF in CD is the utilization of bioprosthetic materials as fillers for fistula tracts (*9*, *24*–*26*). As shown in a previous study (*25*), the approach, however, has multiple complications, including a 20% rate of plug extrusion and a 15% rate of surgical site infection. In addition, the overall healing rate was only 56%. The long-term healing rate is even lower, due to the fact that the recurrence rate is high (*25*, *26*). In summary, the overall success of all current treatments for CD-PAF, including injection of ADSCs into fistula tract, still leaves more than 40% of CD-PAF without adequate treatment (*6*).

On the basis of these studies and our clinical experience, we advance that a systemic treatment targeting luminal inflammation (such as biologics or small molecules) needs to be coupled with a local treatment of CD-PAF for maximum impact on fistula tracts. The ideal local fistula treatment should be applied directly in the fistula tract to address local inflammation, as well as the physical defect that allows drainage of stool, pus, and blood. We designed and tested such a tissue repair scaffold to satisfy multiple conditions: (i) ease of application into the fistula tract, (ii) high porosity to allow regenerative host cell migration into the scaffold, (iii) substantial mechanical stiffness to retain its shape and integrity within the fistula tract, (iv) immunomodulatory properties and enhanced angiogenic responses and blood vessel in-growth into the scaffold, and (v) ability to co-deliver ADSCs and retain them within the composite for maximum ADSC effect within the fistula tract. Building on our previous work and aiming to fulfill these design criteria, we have created a hyaluronic acid (HA) hydrogel covalently linked to electrospun poly(ε-caprolactone) (PCL) nanofiber fragments forming an integrated nanofiber-hydrogel composite (NHC). This NHC is mechanically fragmented (mfNHC) and loaded with ADSCs and then tested in a clinically relevant rat model of CD-PAF.

## RESULTS

### Fabrication of mfNHC with tunable stiffness and pore size

**Fig. 1.**
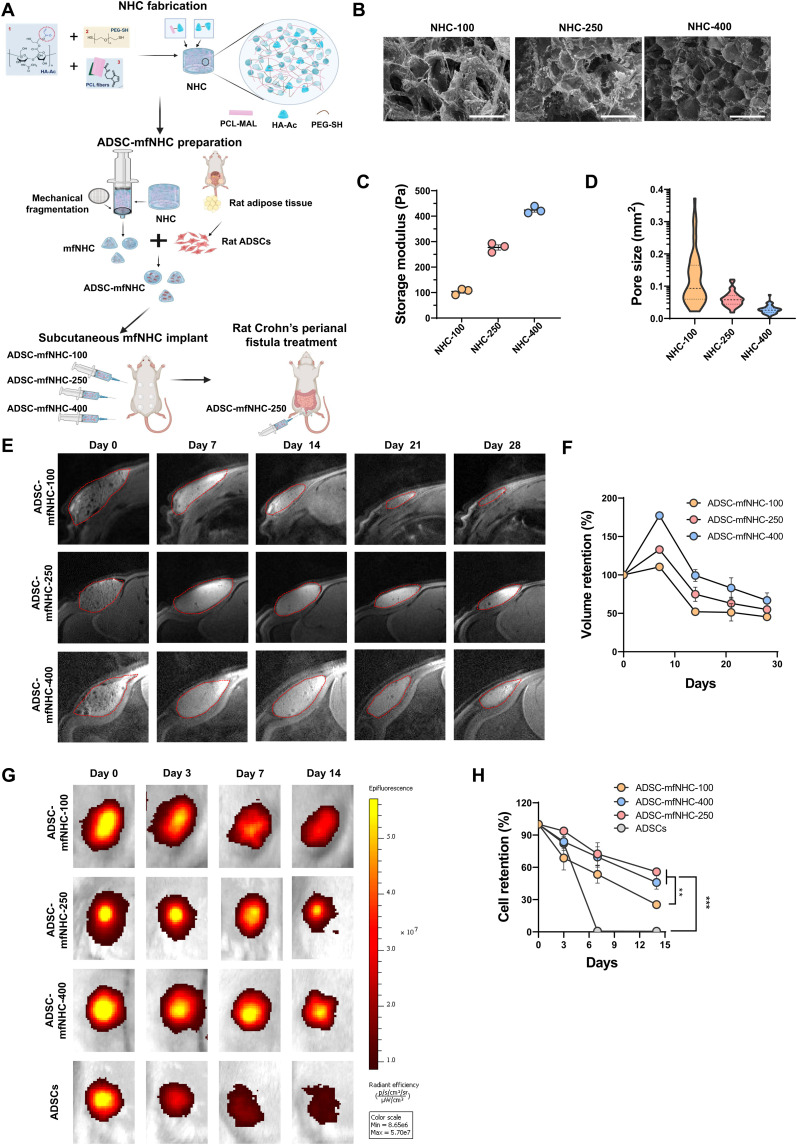
Synthesis and characterization of ADSC-mfNHC. (**A**) Schematic of NHC synthesis and ADSC-mfNHC delivery in a rat model of CD-PAF. (**B**) SEM images of lyophilized NHC at stiffness levels of 100, 250, and 400 Pa, showing microarchitecture of PCL nanofiber-HA hydrogel network with various pore sizes. Note the larger pores in softer mfNHC. (**C**) Measured storage moduli of NHC prepared at different HA concentrations. (**D**) Quantification of the pore size of NHCs with different storage moduli. (**E**) MRI images of mfNHC with different storage moduli loaded with ADSCs over 28 days. mfNHC implants are marked with the red dashed line. (**F**) Quantification of relative ADSC-mfNHC implant volume changes. (**G**) In vivo images of DiR-labeled ADSCs demonstrate retention of ADSCs in the mfNHC implants over a period of 14 days. (**H**) Quantification of relative ADSC retention in mfNHC implants with different moduli (*n* = 4, **P < 0.01, ***P < 0.001).

mfNHC was synthesized by conjugating PCL fiber fragments with HA hydrogel as we previously described (*27*, *28*). For ease of administration, we developed an NHC composite that can be injected into the fistula tract. We designed and synthesized mfNHC of several stiffness levels to identify the optimal material stiffness to allow injection into the fistula tract, as well as host cell infiltration into the composite. First, we tuned the composite pore size, because this mechanical feature directly affects the ability of host regenerative cells to migrate into the composite (*28*). HA was first reacted with glycidyl acrylate to conjugate the acrylate groups to HA to generate an NHC precursor, acrylate-modified HA (HA-Ac). Next, electrospun PCL nanofibers were prepared as described before (*28*). PCL fibers were then surface-activated with plasma treatment to get carboxylic groups on the surface of PCL fiber, followed by converting the carboxyl group to the thiol-reactive maleimide (MAL) group to generate MAL-functionalized PCL (MAL-PCL) fibers. The functionalized PCL fibers were cryo-milled into fragments with length of 40 to 80 μm. NHC was then fabricated by cross-linking HA-Ac, MAL-PCL fibers, and dithiol poly(ethylene glycol) (PEG-SH) at 37°C for 16 hours (Fig. 1A). MAL-PCL fiber fragments and HA-Ac were conjugated to PEG-SH cross-linked network, forming interfacial covalent bonds to generate an integrated composite structure during the gelation process (Fig. 1A). As NHC mechanical properties and pore size are determined by the cross-link network density, we tested multiple HA concentrations of 5, 10, and 15 mg/ml while keeping MAL-PCL fiber content constant. The measured shear modulus *G*′ of NHC stiffness at HA concentration of 5, 10, and 15 mg/ml was 100, 250, and 400 Pa (Fig. 1C). NHC was then mechanically fragmented using our previously reported method (*29*) by passing it through a stainless steel mesh with a uniform pore opening of 0.009 inches to produce mfNHC with an average diameter of 131.3 ± 18.8 μm. As presented in Fig. 1A, the preformed NHC was loaded in two syringes, connected with a union fitted with a stainless steel mesh inside. NHC was sieved by passing through the mesh back and forth twice. The generated mfNHC via this method has an irregular and aspherical shape but with a relatively narrow size distribution (*29*). The microstructure of mfNHC at different shear storage moduli *G*′ (100, 250, or 400 Pa) was analyzed by scanning electron microscopy (SEM). Representative images are shown in Fig. 1B. The SEM image displays the PCL fiber–HA hydrogel network with fibrillary microarchitecture, with various pore sizes as a function of the NHC stiffness. The average pore size was 0.12 ± 0.08 mm^2^ for 100-Pa NHC (NHC-100), 0.06 ± 0.02 mm^2^ for 250-Pa NHC (NHC-250), and 0.03 ± 0.01 mm^2^ for 400-Pa NHC (NHC-400; Fig. 1D). As expected, the pore size of NHC decreased with increasing stiffness. As the concentration of HA increased in NHC, the internal microstructure became more compact, and the pore size decreased. NHC-250 and NHC-400 showed more uniform size distribution compared to NHC-100.

### Isolation and characterization of ADSCs

Rat ADSCs were isolated and characterized for mesenchymal stem cell (MSC) markers (fig. S1A). We found that the isolated cells were positive for mesenchymal markers CD29 (93.7%) and CD90 (99%), but negative for hematopoietic lineage marker CD45 and the endothelial cell marker CD31 (*30*), confirming their ADSC lineage. In addition, these cells expressed CD44 (60.2%), which was reported to be an MSC marker that facilitates cell migration for tissue regeneration via CD44-hyaluronan interaction (*30*). Furthermore, the ADSCs were found to express the MSC marker CD106 (66.4%), which is involved in immunoregulation (*30*). The undifferentiated rat ADSCs showed typical spindle-shaped morphology (fig. S1B). Last, as shown in fig. S1 (C and D), these rat ADSCs demonstrated ability for adipogenesis and osteogenesis as described before (*31*).

### In vitro ADSCs grown in mfNHC at three different levels of stiffness

A total of 0.2 million ADSCs were mixed with 200 μl of mfNHC and cultured for 3 days, and mfNHC-ADSC mixture constructs were collected for frozen sections. As shown in fig. S2A, the confocal images showed that ADSCs were able to adhere and spread into mfNHC to grow in each of the three different stiffness of mfNHC. In addition, to measure the proliferation of ADSCs in mfNHC construct, mf-NHC-ADSCs were seeded into 48-well plates. The cell proliferation was measured for a period of 2 weeks. The cell viability data demonstrated in fig. S2B showed that ADSCs proliferated steadily on mfNHC over 2 weeks, regardless of the stiffness in the range of 100 to 400 Pa.

### In vivo volume retention of injected mfNHC

To evaluate key design features of mfNHC before testing in a PAF model, we implemented a simpler subcutaneous model. This model allows the identification of the optimal stiffness of mfNHC, the measurement of retention of ADSC within NHC, and the extent of host cell infiltration into NHC before testing in the more complex rat model of PAFs in CD. In brief, ADSCs were mixed with mfNHC particles immediately before injection. A total volume of 200 μl of mfNHC, at three different levels of mfNHC stiffness, containing 0.2 million of ADSCs each, was subcutaneously injected into the flank of Sprague-Dawley rats. Magnetic resonance imaging (MRI) scanning was performed, and images were used to reconstruct a three-dimensional (3D) representation of the mfNHC implant at different time points (Fig. 1E). There was swelling (i.e., increase of the volume after injection) within 1 day of injection, and mfNHC with a higher *G*′ resulted in a higher degree of swelling: 77.3 ± 9.7% for 400-Pa mfNHC (mfNHC-400), 33 ± 2.2% for 250-Pa mfNHC (mfNHC-250), and 10 ± 5.0% for 100-Pa mfNHC (mfNHC-100) on day 7. The volume of the construct decreased gradually over 4 weeks. Quantification of the volume of mfNHC-ADSC implants at different storage moduli is shown in Fig. 1F, showing the biggest volume decrease for mfNHC-100 (45.4 ± 2.4%) by day 28 and a similar volume decrease for mfNHC-250 and mfNHC-400 (55.0 ± 3.0% and 57.4 ± 3.7%, respectively).

### Retention of ADSC within mfNHC

It has been hypothesized that the proregenerative action of ADSCs at the site of injury is limited by the ADSC migration away from the injection site (*25*). We postulated that ADSCs injected alone will migrate from the site of injection, while ADSCs delivered embedded into mfNHC will be retained at the site of injection longer. To investigate ADSC retention within mfNHC, we used 1,1′-dioctadecyltetramethyl indotricarbocyanine iodide (DiR)–labeled ADSCs and delivered them embedded in mfNHC subcutaneously in the flank of Sprague-Dawley rats. In vivo fluorescence imaging of DiR-labeled ADSCs at different time points was performed with a PerkinElmer in vivo imaging systems (IVIS) Spectrum system (Fig. 1G). ADSCs injected alone in the subcutaneous space migrated away from the site of injection, as evidenced by the decrease in fluorescence intensity. By day 7, less than 1% of ADSCs were still at the site of injection (0.67 ± 0.06% retained). In sharp contrast, most ADSCs delivered embedded in mfNHC were retained at the site of injection. At day 7, the relative retention rates of ADSCs in mfNHC-400 and mfNHC-250 were 69.3 ± 16.3% and 72.4 ± 18.1% (no statistical difference), respectively, while the retention in mfNHC-100 was lower at 53.4 ± 13.3% (Fig. 1H). The retention of ADSCs at the site of injection slightly decreased at day 14. mfNHC-400 and mfNHC-250 demonstrated similar retention rates (45.9 ± 10.8% and 55.8 ± 5.7%, respectively; no statistical significance), while mfNHC-100 displayed a lower retention rate at 25.4 ± 2.1%.

### Migration of host cells into mfNHC

**Fig. 2.**
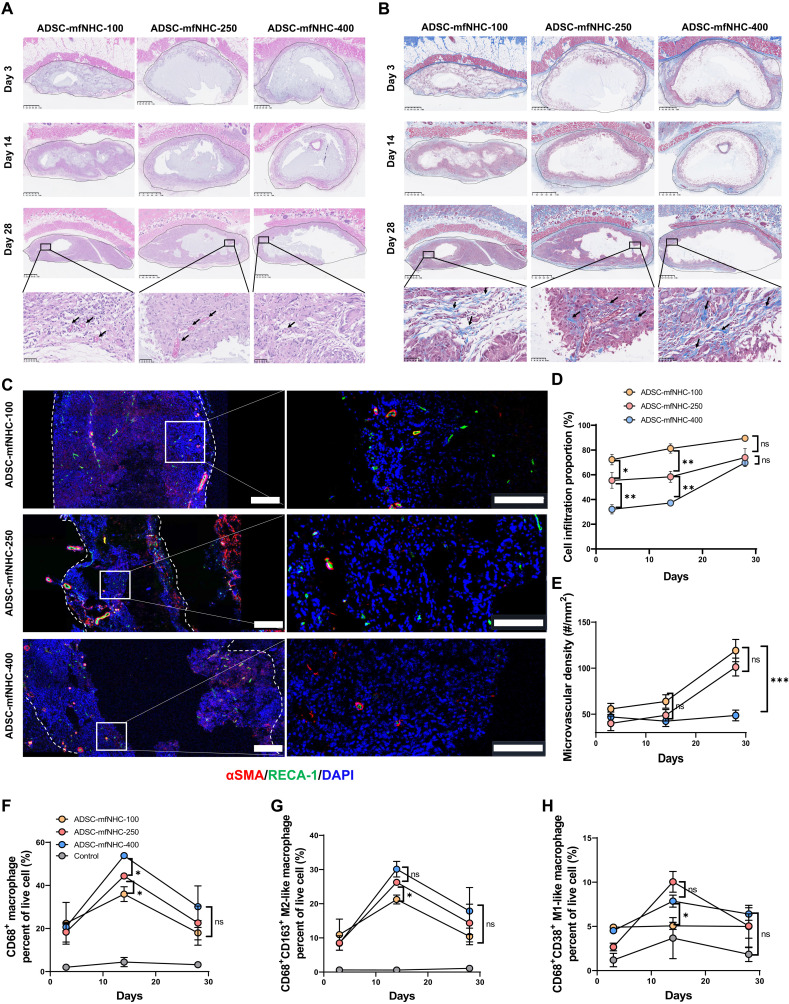
Host cell infiltration, macrophage polarization, and angiogenesis in ADSC-mfNHC implants in a rat subcutaneous injection model. (**A**) Hematoxylin and eosin (H&E) staining of ADSC-mfNHC of three storage moduli at days 3, 14, and 28. The images show host cell infiltration and microvascular formation in the implants (top scale bar, 1 mm; bottom scale bar, 50 μm). (**B**) Masson’s trichrome staining of ADSC-mfNHC with three storage moduli at days 3, 14, and 28. The images show rare collagen deposition in the implants (top scale bar, 1 mm; bottom scale bar, 50 μm). (**C**) IF staining highlights microvascular structures in mfNHC implants. White dashed line demarcates the mfNHC implant. Red, αSMA; green, RECA-1; blue, DAPI (left scale bar, 200 μm; right scale bar, 100 μm). (**D**) Quantification of relative percent of host cell infiltration in the implants of different storage moduli for a period of 28 days (*n* = 3 implants, *P < 0.05, **P < 0.01; ns, no significance). (**E**) Quantification of relative percent of microvascular structures in the implants per square millimeter at different storage moduli for a period of 28 days (*n* = 4 implants, ***P < 0.001). (**F** to **H**) Flow cytometry demonstrates that macrophage infiltration increases from days 3 to 14 and then decreases to day 28. Notably, while the M2 subtype (proregenerative) displays a similar pattern of increase from days 3 to 14 and a decrease to day 28, the M1 subtype (proinflammatory) is constant from days 3 and 14 to 28 (*n* = 4 implants, *P < 0.05).

The mfNHC constructs were harvested at different time points to analyze the degrees of host cell infiltration into the constructs (Fig. 2, A and B). Notably, there was only faint Masson’s trichrome staining in mfNHC (Fig. 2B) indicative of relatively little fibrosis and scar formation. At days 14 and 28, there was evidence of early phase of angiogenesis in mfNHC (Fig. 2, A to C, and fig. S3). As shown in Fig. 2D, at the earliest time point (day 3), there was more host cell infiltration in the softer mfNHC (mfNHC-100: 72.4 ± 7.3%) compared to mfNHC-250 (55.4 ± 11%) and mfNHC-400 (32.1 ± 6.6%). The trend continued to day 7 (mfNHC-100: 81.2 ± 6.6%; mfNHC-250: 58.3 ± 7.7%; and mfNHC-400: 37.2 ± 4.1%). However, at day 28, host cell infiltration levels were comparable for all three groups (NHC-100: 89.4 ± 4%; mfNHC-250: 73.9 ± 12.7%; and mfNHC-400: 69.7 ± 4.1%). We concluded that in the early days after injection of mfNHC, host cells infiltrated faster into mfNHC with a lower stiffness; however, by day 28, host cell infiltration was approximately the same irrespective of mfNHC stiffness.

Angiogenesis is a salient feature of tissue repair and regeneration. To investigate angiogenesis as a function of mfNHC stiffness and ADSC content, we analyzed explanted mfNHC at days 3, 7, and 28. Immunofluorescence (IF) staining for vascular endothelial cell surface marker rat endothelial cell antigen-1 (RECA-1) (*32*) and vascular smooth muscle cell differentiation marker α-smooth muscle actin (α-SMA) (*33*) demonstrated new vessel formation in mfNHC of all three groups (Fig. 2C and fig. S3). At days 3 and 14, there was no difference in the new vessel formation density among the three groups of mfNHC (Fig. 2E). However, by day 28, the softer mfNHC constructs (mfNHC-100 and mfNHC-250) showed a significantly higher blood vessel density compared to mfNHC-400 (mfNHC-100: 119.17 ± 26.97 mm^2^; mfNHC-250: 101.2 ± 15.69 mm^2^; mfNHC-400: 48.67 ± 15.3 mm^2^; Fig. 2E).

Previous studies have shown that macrophage infiltration and polarization correlate with angiogenesis (*28*). Activated macrophages are able to differentiate into different subtypes, including M1- and M2-like macrophages (*34*). CD68, CD38, and CD163 have been well used as markers for pan-macrophage, M1-type–like macrophage, and M2-type–like macrophages, respectively (*35*–*40*). The M1-like (CD68^+^ CD38^+^) cells are associated with a proinflammatory phenotype, while M2-like (CD68^+^ CD163^+^) cells are associated with a proregenerative phenotype that facilitates tissue regeneration (*34*). Single-cell suspension from mfNHC implants at different time points was collected, and flow cytometry was performed to quantify macrophage subpopulations. The flow cytometry results in Fig. 2 (F to H) and figs. S4 to S6 show that the percent of total macrophages (CD68^+^) increased from days 3 to 14 and then decreased at day 28 in each of the three types of mfNHC constructs. Analysis of the macrophage subgroups showed a low percentage of proinflammatory M1-like type at each time point for each of the mfNHC groups. However, the amount of proregenerative M2-like macrophages increased significantly from days 3 to 14 (from 4.8% to 30.1% of live cells) and then decreased from days 14 to 28 (from 30.1% to 13.6% of live cells). Notably, mfNHC-250 and mfNHC-400 showed a similar increase range of M2 cells from days 3 to 14, which was higher compared to mfNHC-100. At day 28, there was no significant difference in percent CD68^+^ macrophages as well as subtypes of CD38^+^ M1-like and CD163^+^ M2-like macrophages across the three groups of mfNHC. These results showed that mfNHC promotes macrophage differentiation into proregenerative M2 subtypes.

Collectively, mfNHC-400 was similar to mfNHC-250 in terms of volume retention and ADSC retention, while mfNHC-100 was less effective. In terms of host cell infiltration and angiogenesis, mfNHC-100 and mfNHC-250 performed better than mfNHC-400. These results led to the choice of NHC-250 as the optimal mfNHC for all subsequent studies.

### Establishment and validation of a rat model of CD-PAF

**Fig. 3.**
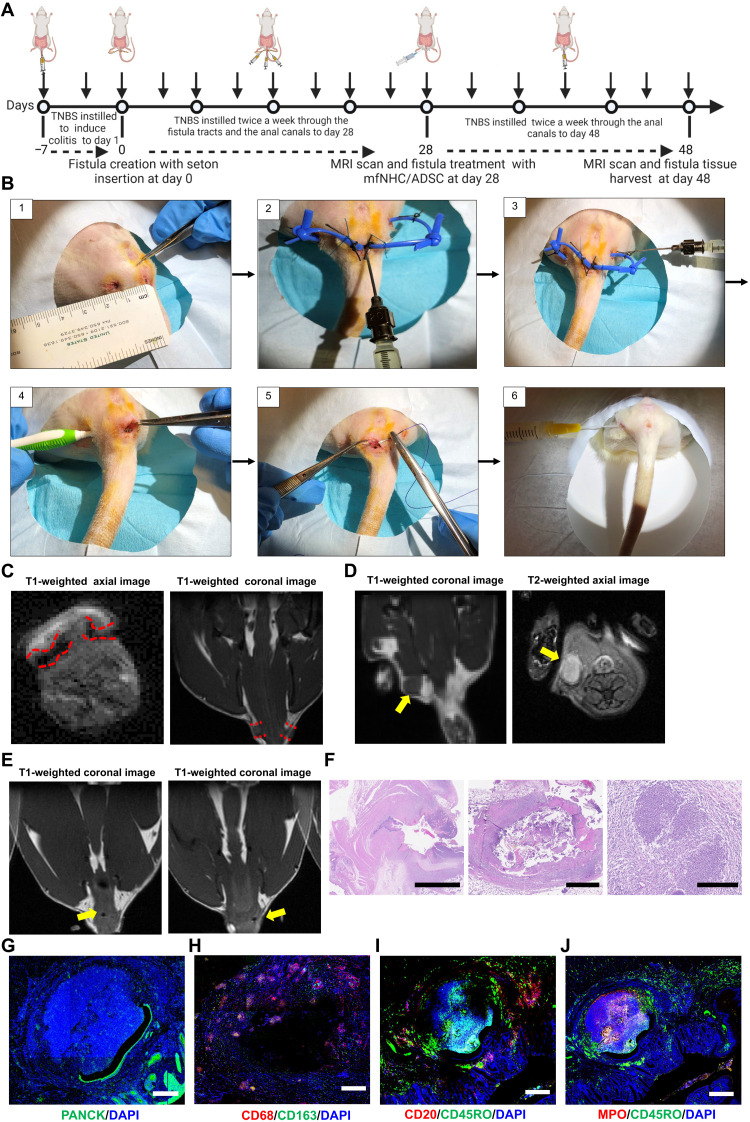
Establishment and characterization of CD-PAF in a rat model. (**A**) Modeling and treatment timeline of CD-PAF in a rat model. (**B**) Steps in rat surgery to induce fistulas and then treat them with mfNHC. (**C**) MRI images at day 28 demonstrating patent fistula tracts (axial T1 images were obtained with setons in place, and coronal T1 images were obtained after setons were cut). (**D**) MRI images showing inflammation around fistula tracts (left, coronal T1, yellow arrow) and occasional abscess formation at day 28 (right, axial T2, yellow arrow). (**E**) Follow-up MRI at day 42 showing patent fistulas (yellow arrow) 14 days after removing setons at day 28. Note that there was no healing and spontaneous closing. (**F**) H&E staining of the fistula tract with a partially re-epithelialized lumen surrounded by dense acute and chronic inflammation and peri-fistula abscess formation (left scale bar, 2.5 mm; middle scale bar, 1 mm; right scale bar, 250 μm). (**G**) IF staining demonstrating PANCK^+^ epithelial cells lining fistula lumens. Green, PANCK; blue, DAPI (scale bar, 200 μm). (**H** to **J**) IF staining demonstrating the spatial arrangement of the inflammatory milieu around the fistula tracts. MPO^+^, neutrophils; CD68^+^CD163^+^, macrophages; CD20^+^, B cells; CD45RO^+^, memory T lymphocytes (scale bar, 500 μm).

The human CD-PAF fistula tract environment is inflammatory and antithetical to healing. We modified a previously published PAF protocol in a rat model (*41*) to closely mimic this proinflammatory milieu. In brief, six rats were treated with 2,4,6-trinitrobenzenesulfonic acid solution (TNBS) to induce colitis (Fig. 3, A and B). All rats developed clinical symptoms of proctitis/colitis, including soft feces, diarrhea, blood per rectum, anorectal mucosa swelling, and mucosa bleeding with light touch. Rats started to develop symptoms after two doses of TNBS. Fistulas were created surgically at the 3 o’clock and 9 o’clock positions. The exit points of the fistula tracts in the perianal skin were chosen approximately 1 cm away from anus. Setons were placed in the newly created tracts to keep them open. We modified the protocol reported by Flacs *et al.* (*41*) to increase the severity of fistulas. Specifically, we used a thicker seton (1.2 mm × 2.4 mm in cross section) with a larger inner diameter, which translates into a more difficult to treat model. In addition, to maintain an inflammatory milieu, after fistulas were treated at day 28, we continue to instill 5% TNBS in the rectum of the rodents, twice a week, until the end of the experiment. Throughout the period of irrigation of fistulas with TNBS, the rats continued to have clinical signs of ongoing rectal inflammation (mucosa swelling, soft stools, diarrhea, and blood per rectum), which is desirable in a model of CD-PAF. After 4 weeks of TNBS irrigation of fistula tracts, we performed MRI on all animals to evaluate the degree of fistula formation. As shown in a representative image in Fig. 3C, two large patent tracts on the left and right side of perianal area were imaged at day 28. In addition, there was evidence of inflammation around the fistula tracts and occasional abscesses were found around fistulas (Fig. 3D). The setons were removed at day 28. Repeat MRI showed that the fistulas were still patent at day 42, which indicates that fistulas have matured and did not have the tendency for spontaneous healing (Fig. 3E). At day 42, the animals were sacrificed, and fistula tissues were harvested for histopathology. Hematoxylin and eosin (H&E) staining demonstrated that the fistula tracts were partially re-epithelialized and were surrounded by marked acute and chronic inflammation, granulation tissue, and mild to moderate fibrosis. Patchy foreign body giant cell type reaction was also present, as were occasional peri-fistula tract abscesses. Intraluminal fecal material was also identified (Fig. 3F). IF staining demonstrated that fistula lining of flattened pan-cytokeratin (PANCK)–positive intestinal epithelial cells likely migrated from the intestinal side of the fistula or narrow squamous epithelium likely migrated from the cutaneous side of the fistula (Fig. 3G). We further characterized the inflammatory milieu within the fistula tracts using IF staining for MPO^+^ neutrophils, CD68^+^ and CD163^+^ macrophages, CD20^+^ B cells, and CD45RO^+^ memory T lymphocytes (Fig. 3, H to J).

### Treatment efficacy using mfNHC-250 in a rat model of CD-PAF

**Fig. 4.**
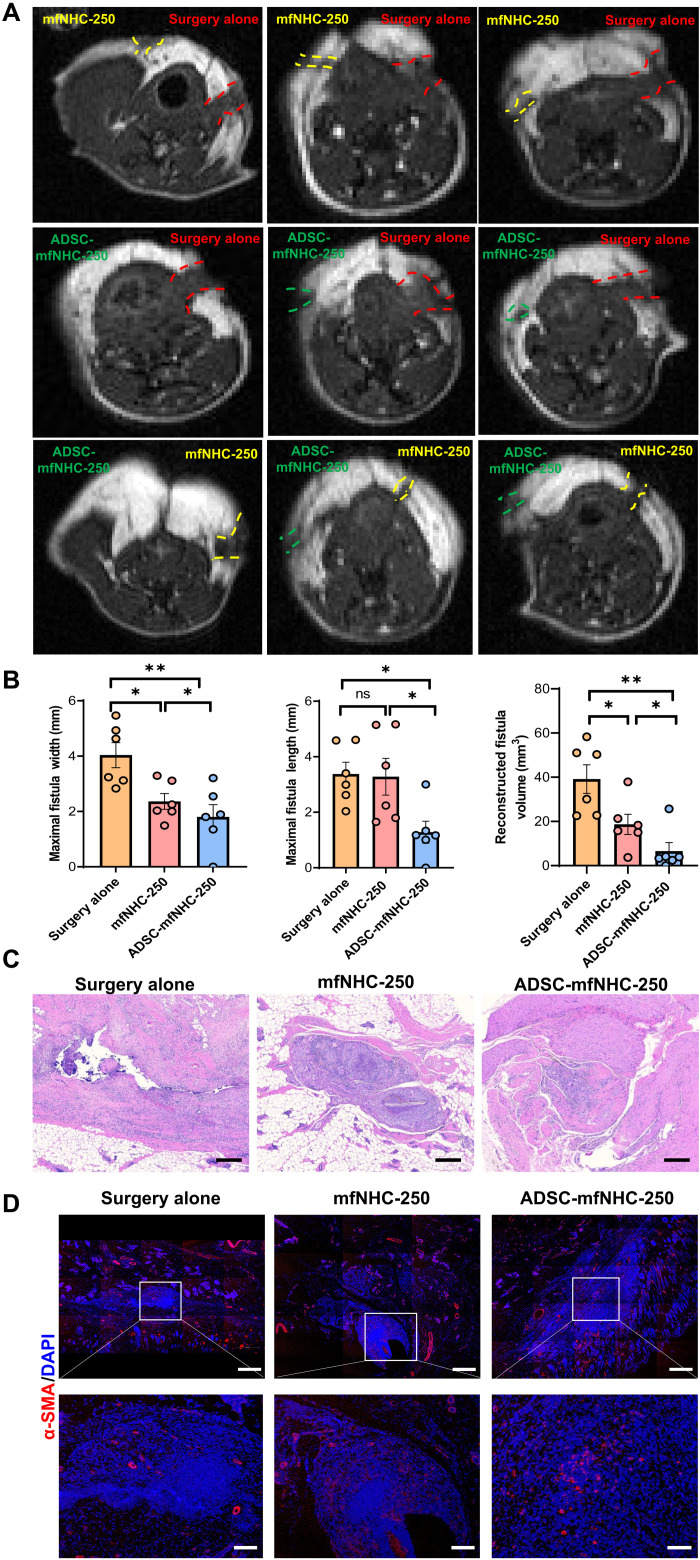
Effectiveness of ADSC-mfNHC-250 in treating CD-PAF in a rat model. (**A**) MRI images demonstrating that ADSC-mfNHC-250 promotes the best healing of fistulas (green dashed line) compared with mfNHC-250 (yellow dashed line) and surgery at day 48 (red dashed line). (**B**) Measurements of the fistula tracts and quantification of reconstructed fistula 3D volumes from each treatment group (*n* = 6, *P < 0.05, **P < 0.01, ***P < 0.001). (**C**) H&E staining of fistulas after treatment. Note that ADSC-mfNHC-250 treatment resulted in a marked reduction in acute and chronic inflammation as compared to mfNHC-250 treatment or surgical treatment (scale bar, 200 μm). (**D**) IF staining showing microvasculature in fistulas with three different treatments. Red, α-SMA; blue, DAPI (top scale bar, 500 μm; bottom scale bar, 100 μm).

mfNHC-250 was evaluated for its healing capabilities in a rat model of CD-PAF. To minimize the individual variations from animal to animal, we elected to treat one of the fistulas and assign the other one in the same animal as the surgical control. Both the control and mfNHC treatment fistulas included surgical closure of the internal orifice. mfNHC is inserted into the treated fistulas through the skin orifice of the fistulas after closure of the internal orifice. Therefore, mfNHC was well retained within the fistula tract of the treated tract. Rats were imaged with MRI 20 days after fistula treatment with mfNHC-250. Both axial and coronal T1-weighted perianal MRI scanning was carried out. As shown in Fig. 4A, T1-weighted images indicated that fistulas treated with ADSC-mfNHC-250 healed faster when compared to mfNHC-250 alone (without ADSC) and when compared to surgery alone. Two of six fistulas in the ADSC-mfNHC-250 treatment group showed complete healing, and the other four displayed more than 80% healing with closure of the internal orifice and/or external orifice. In the mfNHC-250 group, there was moderate fistula healing with partial internal orifice closure. The external orifices remained open; in addition, fistula tracts were larger compared to ADSC-mfNHC-250 group. In the surgery group, fistulas showed the least healing, with large fistula tracts and both internal and external orifices open. All axial and coronal MRI scanning images are shown in figs. S7 to S15. Movies S1 and S2 show a 3D reconstruction of representative fistulas. Figure 4B contains dot plots comparing fistula length, width, and volume among the two treatment arms (ADSC-mfNHC-250 and mfNHC-250) and the surgery arm. The calculated volume of fistula tracts was 39.18 ± 15.8 mm^3^ for the surgery group, 18.67 ± 11.16 mm^3^ for the mfNHC-250 group, and 6.57 ± 9.5 mm^3^ for the ADSC-mfNHC-250 group.

H&E staining confirmed that both surgically treated fistulas and fistulas treated with mfNHC-250 had acute and chronic inflammation, foreign body giant cell response, and occasional peri-fistula abscess formation. Fistulas treated with mfNHC-250 had only moderate amounts of acute and chronic inflammatory cells present, while surgically treated fistulas had severe inflammation. In stark contrast, the ADSC-mfNHC-250–treated fistulas displayed only mild chronic inflammation with an associated foreign body giant cell reaction (Fig. 4C). In addition, IF staining showed that neovessel formation was most pronounced in the ADSC-mfNHC-250–treated fistulas (Fig. 4D and fig. S16). These results indicated that ADSC-mfNHC-250 induces the highest degree of fistula closure, the least inflammatory infiltrate, and the most vigorous angiogenesis.

### Effect of ADSC-mfNHC-250 treatment on gene expression

**Fig. 5.**
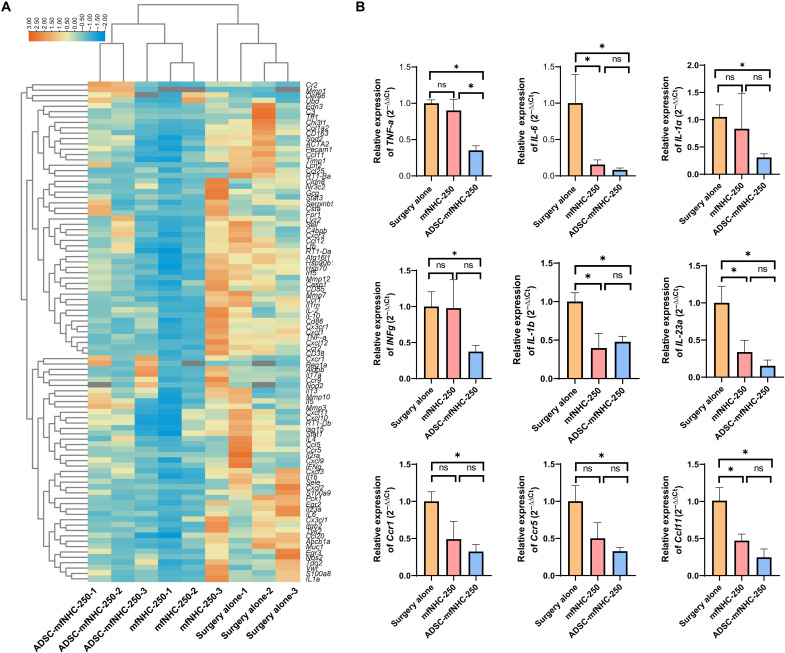
RT-PCR array with RNA from treated fistulas. (**A**) Heatmap of a panel of genes relevant to CD. (**B**) RT-PCR of select genes from the RT-PCR panel confirmed that ADSC-mfNHC-250 inhibited critical proinflammatory cytokine transcripts that participate in the inflammatory cascade, such as TNF-α (*TNF-a*), IL-6 (*IL-6*), IL-1α (*IL-1a*), IFN-γ (*IFNg*), IL-1β (*IL-1b*), IL23α (*IL23a*), Ccr1 (*Ccr1*), Ccr5 (*Ccr5*), and Ccl1 (*n* ≥ 3, *P < 0.05).

To explore the mechanisms through which ADSC-mfNHC-250 promotes fistula healing, we performed real-time polymerase chain reaction (PCR) arrays on RNA extracted from the PAF tissues. We used a reverse transcription PCR (RT-PCR) array kit containing genes relevant to CD-PAF. As shown in Fig. 5A, ADSC-mfNHC-250 treatment inhibited genes relevant to inflammation and immunity: Ccl11, Ccr2, Ccr5, Ccr9, interferon-γ (IFN-γ), interleukin-13 (IL-13), IL-5, IL-6, and extracellular matrix– and fibrosis-related genes: Cx3cr1, Cxcl12, Cxcr3, ITGB2, Mmp1, Mmp10, Mmp12, Mmp3, and Mmp7. Real-time RT-PCR for selected genes from the RT-PCR array validated these findings. Specifically, ADSC-mfNHC-250 inhibited TNF-α (2.85-fold), IL-6 (16.7-fold), IL-1α (3.2-fold), IFN-γ (2.7-fold), IL-1β (2-fold), IL-23α (6.7-fold), Ccr1 (3.1-fold), Ccr5 (3-fold), and Ccl1 (4-fold), compared to surgical treatment (Fig. 5B). mfNHC-250 alone inhibited only four of these nine genes: IL-6 (6.7-fold), IL-1β (2.5-fold), IL-33 (3-fold), and Ccl11 (2.1-fold). These results suggested that ADSC-mfNHC-250 inhibits the proinflammatory and profibrotic milieu of PAFs to induce tissue repair.

### Effect of ADSC-mfNHC-250 treatment on immune cell infiltration

**Fig. 6.**
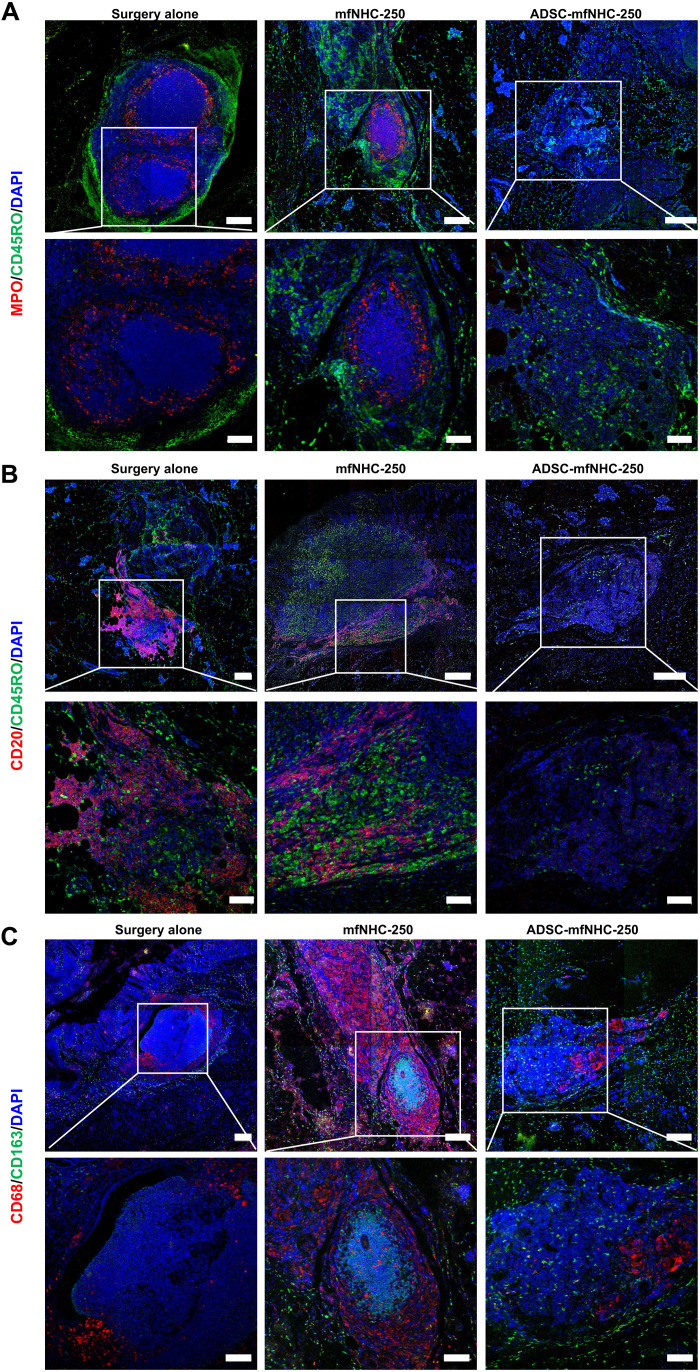
IF staining of fistulas. (**A**) Staining of MPO^+^ neutrophils and CD45RO^+^ T cells. (**B**) Staining of CD20^+^ B cells and CD45RO^+^ memory T cells. (**C**) Staining of CD68^+^ macrophages and CD163^+^ M2-like macrophages (top scale bar, 200 μm; bottom scale bar, 100 μm).

**Fig. 7.**
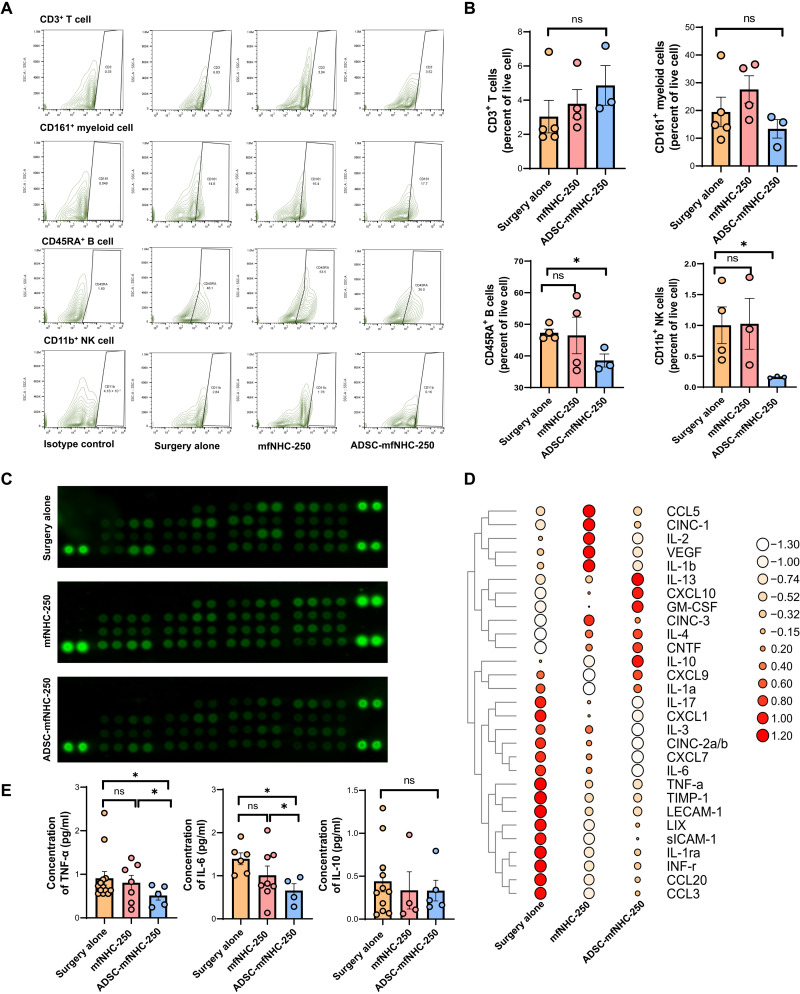
Flow cytometry analysis of immune cell distribution and cytokine profile analysis of CD-PAF treated with ADSC-mfNHC-250. (**A**) Flow cytometry contour plot of a general panel of T cell, B cell, NK cell, and myeloid profiles with CD3, CD45RA, CD11b, and CD161. (**B**) Flow cytometry analysis indicated that ADSC-mfNHC-250 inhibited local CD11b^+^ and CD45RA^+^ immune cell expression, but there is no difference in CD3^+^ T and CD161^+^ myeloid cell profiles (*n* ≥ 3, *P < 0.05). (**C**) Cytokine profile array plot images for fistula with different treatments. (**D**) Heatmap of cytokine profile array quantification showed that ADSC-mfNHC-250 inhibited several proinflammation cytokines such as IL-6, TNF-α, IL-17, CCl3, and IL-1ra compared with mfNHC-250 treatment as control surgery alone treatment. In the meantime, ADSC-mfNHC-250 increased anti-inflammation cytokines IL-10 and IL-4 compared with the remaining two treatment groups. (**E**) ELISA confirmed that ADSC-mfNHC-250 inhibited two vital proinflammation cytokines TNF-α and IL-6 in CD in larger-scale samples. However, there is no significant difference of the anti-inflammation cytokine IL-10 in all treatment groups (*n* > 4, *P < 0.05).

To investigate the impact of treatment on local immune cells, we performed IF staining on fistula tissue in all treatment groups (*41*, *42*). As shown in Fig. 6 (A to C), ADSC-mfNHC-250–treated fistulas had relatively few MPO^+^ neutrophil cells, CD20^+^ B lymphocytes, CD45RO^+^ memory T lymphocytes, and CD68^+^ macrophages compared with the surgery fistulas and mfNHC-250–treated fistulas. To further explore local immune cell distribution across the three treatment groups, fistula tissue was collected at the end of each treatment group, dissociated into single-cell suspension, and analyzed by flow cytometry. As shown in Fig. 7 (A and B), there were fewer CD11b^+^ natural killer (NK) and CD45RA^+^ B cells in ADSC-mfNHC-250–treated fistulas. These results suggested that ADSC-mfNHC-250 decreases local inflammation and inflammatory cells and shifts the local milieu toward regeneration and healing.

### Effect of ADSC-mfNHC-250 treatment on cytokine secretion within the fistula tracts

Cytokine arrays were used with protein extracts from fistula tissues. Figure 7C shows the protein array blot image of 29 cytokines. Figure 7D shows the quantitative results from each treatment blot. The data showed that ADSC-mfNHC-250 and mfNHC-250 inhibited key proinflammatory cytokines such as IFN-γ, CCL3, IL-6, TNF-α, CCL20, and IL-17 and increased anti-inflammatory cytokines such as IL-4 and IL-13. To further confirm the CD-PAF–related cytokine changes, enzyme-linked immunosorbent assay (ELISA) was performed with proinflammatory cytokines TNF-α and IL-6 and anti-inflammatory cytokine IL-10. The results in Fig. 7E showed that ADSC-mfNHC-250 inhibited TNF-α (2-fold) and IL-6 (1.5-fold) compared to the surgery-treated fistulas. There is no significance difference, however, between mfNHC-250 treatment and surgery-treated fistulas. The results indicate that ADSC-mfNHC-250 modulates cytokine secretion of immune cells such as helper T cells and macrophages, with the net effect of acceleration of fistula healing.

## DISCUSSION

There are no uniformly effective treatments for PAFs in CD (*10*). A variety of treatments are currently used clinically or investigated in human clinical trials. These treatments include biologic and small molecule for the treatment of luminal CD and placement of setons to improve drainage and reduce inflammation within fistula tracts. In addition, fistula plugs, ADSC injection around the fistula tracts, and other treatments have been attempted. The overall efficacy of any given treatment, however, is generally less than 50% (*6*, *9*–*20*). There are several reasons for which CD-PAF are extraordinarily difficult to treat. First, fistula tracts arise in an inflammatory millieu, both luminally in the rectum and within the fistula tract itself. The biologic treatments for CD have 50% or less efficacy for luminal gut inflammation and even less for inflammation in the fistula tracts. The placement of setons within the fistula tracts is done to improve pus drainage and prevent abscess formation. The intense inflammation within the fistula tract and the overall exaggerated inflammatory milieu is adversarial to tissue regeneration and healing. Second, fistula tracts communicate with the rectum and, therefore, soft or liquid stools can contaminate the fistula tracts, further complicating any prospect of healing. Surgical closure of the fistula tract, therefore, is an intuitive approach. The current surgical approaches, however, are marred by a high rate of failure and a high rate of fistula recurrence. The reason is believed to be, in part, the ongoing inflammatory process in the rectum that initially contributed to the emergence of fistula and will participate in its recurrence. In addition, from an interventional perspective, PAFs are difficult to address surgically, as the risk of damage to anal sphincter and incontinence is high. Third, fistula tracts are localized in an area of high mechanical forces because of the close proximity to the anal sphincter. Because of these high mechanical forces, any fistula plug material needs to be sufficiently stiff to fill the fistula tract to repair the physical defect and resist expulsion from the fistula tract, but sufficiently soft to allow migration of regenerative host cells into the plug. The challenges with the development of a successful tissue regenerative fistula treatment are immense, and the limited success to date irrespective of approach is testament to these challenges.

A local approach to fistulas in Crohn’s is essential, as systemic anti-inflammatory treatments tend to address luminal inflammation, with minimal impact onto fistula draining and healing. In addition, the communication with the rectum and drainage of stool needs to be interrupted to create a sterile environment and decrease further inflammation so that the fistula can heal. However, surgical closure of fistulas coupled with best treatments for Crohn’s inflammation is not effective except in a small minority of patients. In addition, while stem cell injection around fistula tracts appears in clinical trials to help with local healing of fistulas, as we have shown here, it is unlikely for the stem cells to be retained around the fistula tracts for any meaningful duration of time. As shown in a clinical trial, there was a statistically significant 17.6% points improvement in fistula healing from 41.6% in fistulas treated with curettage and closure of internal orifice alone to 59.2% in fistulas treated similarly, plus injection of ADSC (*6*). The optimized ADSC-mfNHC-250 treatment here showed a sixfold reduction in overall fistula volume (Fig. 4B).

A scaffold that would retain stem cells at the site of fistulas is not currently in clinical use. On the basis of all fistula treatments approved to date, as well as our clinical expertise, an ideal local treatment (i) would surgically close the internal orifice of the fistula as to interrupt the ongoing contamination with stool and bacteria and ensuing inflammation, (ii) would fill the physical defect of the fistula tract, (iii) would retain ADSCs within the fistula tract, (iv) is easily penetrated by proregenerative host cells, and (v) is easily deliverable from a procedural perspective. The data presented here support the hypothesis that the mfNHC we developed, when delivered together with ADSCs, satisfies these requirements. The microparticulate shape and viscoelastic properties of mfNHC match the surrounding soft tissue properties (*43*, *44*), ensuring that (i) the track is fully filled, (ii) the composite conforms to the defect shape of the fistula lumen, and (iii) the composite maintains the integrity of the repair site. Results presented here highlight the advantages of using mfNHC to enhance the retention of the delivered ADSCs inside the tissue repair location, i.e., CD-PAF, for an extended period of time. These results demonstrate a strong effect of mfNHC in healing fistulas in a relevant rat CD-PAF model. Last, the mechanical fragmentation allows for ease of injection into the fistula tract, which is procedurally facile and effective at filling the fistula tracts of various sizes and shapes. Further clinical studies are warranted to further tune this mfNHC and test its proregenerative capacities in human clinical trials.

## MATERIALS AND METHODS

### NHC preparation and rheological assessment

HA-Ac was formulated by mixing 1% sodium hyaluronate (HA; 1.5 × 10^6^ Da; LifeCore Biomedical, Chaska, MN) in PBS (pH 8.5) with glycidyl acrylate at a ratio of 100:3 (v/v) (TCI, Portland, OR) on a magnetic stirrer at 37°C for 16 hours. The modified HA-Ac was precipitated by adding the reaction mixture into ethanol at a volume ratio of 1:10. The precipitate was washed with ethanol and acetone three times each and dehydrated with compressed air. The modified HA-Ac precipitate was then redissolved into PBS (pH 7.4) and stored at 4°C before use.

The electrospun PCL nanofiber fragments were made as described before (*27*, *28*). Briefly, PCL was dissolved in a dichloromethane and dimethylformamide mixture (9:1, v/v) to obtain the PCL solution (16%, w/w). The PCL solution was electrospun to obtain nanofibers, which were treated with plasma to generate carboxylic functional groups on the PCL fiber surface. The carboxylic functional groups were activated and converted to MAL groups by treatment with ethyl dimethylaminopropyl carbodiimide and *N*-hydroxysuccinimide at a molar ratio of 1:4:4, respectively. *N*-(2-Aminoethyl) MAL was added at a molar ratio of carboxyl groups to amine groups of 1:2, and the mixture was gently shaken to facilitate conversion. The MAL-modified PCL fibers were broken into fragments with an average length of 20 to 100 μm using a cryogenic mill (Freezer/Mill 6770, SPEX SamplePrep, Metuchen, NJ). These fragments were sterilized in three cycles of 70% (v/v) ethanol followed by distilled water. The sterilized MAL-modified fiber fragments were lyophilized and stored at −20°C before use.

NHCs with different stiffnesses were formulated by mixing MAL-fibers in HA-Ac precursor solution with PEG-SH (molecular weight: 5 kDa, JenKem Technology, Plano, TX). The composites were prepared with MAL-fibers (30 mg/ml) and varied concentrations of HA-Ac (5, 10, or 15 mg/ml) in PBS (pH 7.4). The concentration of PEG-SH in each formulation was determined by keeping the thiol-acrylate ratio as 1.0, which was 3.6, 6.5, and 9.4 mg/ml corresponding to 5, 10, and 15 mg/ml of HA-Ac. During the gelation process, MAL-PCL fiber fragments were conjugated to PEG-SH, forming interfacial covalent bonds and generating an integrated composite structure with HA-Ac (Fig. 1A). A rheometer (ARG2, TA Instruments, New Castle, DE) was used to measure *G*′ of the composites and hydrogels. The rheological characterization of composites was performed as reported previously (*27*, *28*).

### Scanning electron microscopy

Micrographs of NHC with three storage moduli were obtained using an SEM (Thermo Fisher Scientific Helios G4 UC, USA) and used to assess pore size distribution. MAL-PCL fibers and the gel precursors were cast into an 8-mm poly(dimethylsiloxane) mode. After cross-linking for 16 hours, NHC was lyophilized at −20°C for 24 hours. The cross section of NHC was imaged using SEM at an accelerating voltage of 5 kV. NHC pore size distribution was assessed using ImageJ Fiji [National Institutes of Health (NIH), Bethesda, MD]; at least 50 random pores per condition were assessed.

### In vitro culture of ADSCs with mfNHC at three different levels of stiffness

For in vitro culture, the PCL nanofiber of NHC was labeled with a green fluorescent dye, poly(9, 9-dioctylfouorene-alt-benzothiladiazole) (F8BT), during the mfNHC fabrication (*27*, *28*). One million ADSCs were mixed with 1 ml of mfNHC, and 200 μl of the mixture was seeded into each well of a 0.4-μm filter–fitted 24-well transwell plate (3470, Corning, Kennebunk, ME). Dulbecco’s modified Eagle’s medium (DMEM) high glucose medium supplemented with 10% fetal bovine serum (FBS) was added; 3 days later, ADSCs and mfNHC constructs were collected together and embedded into OCT for frozen sections. The 30-μm section slices were made and fixed with 10% formalin for 10 min and washed with PBS for three times, and the nuclei of ADSCs were stained with DAPI (4′,6-diamidino-2-phenylindole dihydrochloride; 2 μl/ml; Thermo Fisher Scientific) for 10 min at room temperature). The slices were washed with PBS for three times and covered with glass slips in anti-fade fluorescent mounting medium (Dako). Images were then taken with the Zeiss LSM510 Meta Confocal Microscope (Thornwood, NY). In a separate experiment to quantify cell viability, 200 μl of mfNHC was mixed with 10^5^ ADSCs suspended in 100 μl of medium and seeded into each well of a 48-well plate. The cell proliferation was measured with AlamarBlue cell viability reagents (DAL 1025, Thermo Fisher Scientific, Waltham, MA) over 14 days.

### Animal studies

Sprague-Dawley female rats (180 to 220 g) were purchased from Charles River Laboratories (Frederick, MD) and housed in the animal facility at Johns Hopkins University. All animal experiments were approved and performed under the guidelines of the Institutional Animal Care and Use Committee at Johns Hopkins University.

### Rat ADSC isolation and culture

Rat ADSCs were isolated from Sprague-Dawley rats as described previously (*31*). Briefly, rat fat pads were minced and washed three times to eliminate hematopoietic cells and debris. The tissue fragments were then digested with collagenase IV (2.5 mg/ml) in DMEM for 1 hour in a 37°C water bath. Single cells were collected and plated in 24-well cell culture plates. Cells were cultured in DMEM high glucose with 1% penicillin-streptomycin and 10% FBS in 37°C humid cell culture hood supplied with 5% CO_2_. Flow cytometry analysis with ADSC-specific markers was used to confirm ADSCs at passages 3 to 4. ADSCs at passages 4 to 6 were used in the study.

### Subcutaneous mfNHC implantation

Rats were sedated with 2 to 5% isoflurane. Hair at the injection site was removed with clippers, and mfNHC of different stiffnesses (100, 250, and 400 Pa) each mixed with 1 million/ml ADSCs or ADSCs alone was subcutaneously injected into the lateral flank of the rats. Implants were collected at days 3, 7, 14, and 28 for histological assessment and molecular analysis.

### In vivo imaging of DiR-labeled ADSCs imbedded delivered with NHC

To evaluate the impact of mfNHC stiffness on ADSC retention within the composite and distribution of ADSCs in vivo, 1 million rat ADSCs were labeled with DiR (125964, PerkinElmer, Waltham, MA) and mixed with 200 μl of NHC at 100, 250, and 400 Pa, respectively. Specifically, we chose DiR as a lipophilic, cell membrane staining agent because of its stability and low likelihood of cell-to-cell transfer (*45*). In addition, the DiR dye is near-infrared, which reduced the interference from autofluorescence signals from animal, making the quantification more specific. Moreover, it has been reported that the fluorescence signal from the DiR-labeled cells disappears 24 hours after cell death (*46*), supporting that the quantitative analysis of cell retention is largely representative of live cells over 15 days, even considering some variations due to cell death during this period. The mixture grafts were subcutaneously injected into the lateral flank of Sprague-Dawley rats. A volume of a 200-μl rat ADSCs in PBS was subcutaneously injected as a control. An IVIS (Perkin Xenogen IVIS 200 Imaging System Pred Spectrum, PerkinElmer, Waltham, MA) was used to track DiR-labeled ADSCs at days 0, 3, 7, and 14. The fluorescence intensity of DiR-labeled ADSCs was recorded and quantified with a live imaging software (Caliper LifeSciences, Waltham, MA) to reflect the relative retention of rat ADSCs in NHCs.

### MRI of implanted mfNHC

To monitor the subcutaneous mfNHC volume changes at different time points, MRI (Bruker Biospin Corp., Billerica, MA) scanning was used to scan the injected mfNHC composite at different time points. First, rats were sedated and set in a prone position. A T2-weighted turbo spin sequence with fat suppression protocol was applied. Images were captured at an acquisition of 3.5 ms and encoding of 0.3 ms; the field of view (FOV) was 55 mm × 55 mm covering 50-mm length area with 1 mm of thickness per slice for a total of 51 slices. ImageJ Fiji (NIH, Bethesda, MD) was used to analyze the mfNHC volumes over 28 days. There was a swelling of the injected mfNHC within 1 day of the injection. The degree of swelling is defined by percent volume increase over the injected volume.

### PAF establishment

We adapted a published protocol (*41*) to model CD-PAF in rats. In brief, 500 μl of 50% TNBS solution in ethanol (picryl sulfonic acid, Millipore Sigma, Burlington, MA) was given intrarectally to rats twice a week for 1 week to induce proctitis as reported before (*47*). This model is well established and found to be relevant to the biology of IBD patients (*48*). During the PAF establishment surgery, the rats were sedated with 2 to 5% isoflurane and a 14-gauge guide needle was inserted from inside the rectum toward the skin at the 3 o’clock and 9 o’clock location. A suture was then inserted through the plastic sheet of the needle (with the needle removed) into the fistula tract, and a 1.2 mm × 2.4 mm vascular seton (Keysurgical, Eden Prairie, MN) was inserted into the fistula tract. The fistula length was 1 cm (from skin to rectum). TNBS was administrated into the fistula tracts twice a week for 1 month. One hundred microliters of 5% TNBS in 50% ethanol was instilled into each fistula track from outside toward the rectum, and 200 μl is then injected into the rectum. The rat was then positioned upside down for 0.5 to 1 min so that the TNBS is allowed time inside the rectum. After 4 weeks, the PAF MRI was conducted, and tissue was collected for pathology examination to confirm the success of CD-PAF modeling.

### Surgical treatment of fistulas

Four weeks after the fistulas were created, animals were sedated and setons were removed. The fistula tracts were washed with saline. Small toothbrushes (proxabrush, Sunstar Americas, Schaumburg, IL) were used to clean the fistula tracts, as it is done during surgery in patients. Rats were then randomized to receive one of three treatments: (i) surgery alone (brushing of fistula tract followed by closure of internal orifice with 6-0 vicryl suture), (ii) surgery as above plus mfNHC-250 injection into fistula tract, and (iii) surgery alone plus ADSC-mfNHC-250 injection into fistula tract. Specific details of fistula treatment groups are shown in table S1. The timeline is shown in Fig. 4 (A and B). TNBS was instilled through the rectum twice a week to maintain luminal inflammation to closely mimic the environment in CD, until the end point of the study. MRI images were collected at day 20 after surgery to evaluate the effects of the treatment. In our clinical experience, therapeutic failures in the surgical treatment of fistulas in Crohn’s patients are recognized within a matter of days due to ongoing or recurrence drainage of pus, blood, or stool through the fistula tract. Therefore, we decided that follow-up for 20 days is appropriate to evaluate for early healing of fistulas. Tissues were collected for pathology and molecular analysis after MRI image acquisition.

### MRI scanning of fistulas

MRI was used to monitor fistula formation and healing with T1-weighted imaging. Rats were sedated with 2 to 5% isoflurane, and animals were placed in a supine position in a homebuilt enclosure with 9.7-cm-diameter coil with PAF located at the center. Images were acquired with a 9.4-T horizontal Bruker spectrometer (Bruker Biospin Corp., Billerica, MA). For each rat, we acquired a triplanar scout scan, followed by axial T1 images and coronal T1 images. The T1 images were acquired with a Rapid Acquisition with Relaxation Enhancement (RARE) sequence (echo time: 8.2, repetition time: 566, rare factor: 2, FOV: 5 cm × 5 cm, slice thickness: 1 mm), covering 30-mm length area from anus. Fistula length and width at each slice as well as fistula volume were analyzed with ImageJ Fiji (NIH, Bethesda, MD).

### Real-time RT-PCR arrays

PAF tissue from different treatment groups was collected, and 1 ml of TRIzol (Thermo Fisher Scientific, Waltham, MA) was used to lyse the tissue. RNA was extracted following the standard TRIzol Protocol. The RNA was purified with an RNeasy Mini kit (Qiagen, Hilden, Germany) and reversed using the Revert Aid First Strand cDNA Synthesis Kit (K1622, Thermo Fisher Scientific). Real-time RT^2^ Profiler PCR Array Rat CD array (Qiagen, Hilden, Germany) was used to analyze the gene expression profiles. Real-time PCR was carried out with a QuantStudio 3 PCR machine (Applied Biosystems, Waltham, MA). The housekeeping gene GAPDH (glyceraldehyde-3-phosphate dehydrogenase) was used to normalize gene expression. Melting curve analysis was used to confirm the real-time PCR array results.

### Histological assessment

Fresh subcutaneous mfNHC implants and PAF tissue were collected and fixed with 10% neutral formalin for 24 hours. The tissue was set in a cross-resection position in paraffin. Four-micrometer-thick slices were prepared in the Johns Hopkins Hospital Pathology Laboratory. H&E and Masson’s trichrome staining were performed, and slides were analyzed by an experienced gastrointestinal pathologist at the Johns Hopkins Hospital.

### IF staining

Paraffinized sections were deparaffinized, and citrate buffer was used for antigen retrieval following standard protocols. Sections were then washed with PBS three times, permeabilized with 0.1% Triton X-100 in PBS, blocked with 10% goat serum, and stained with first antibodies (showed below) overnight in 4°C. Second antibodies with Alexa fluorescence (Invitrogen, Waltham, MA) were added as described before (*43*). DAPI was used to stain nuclei. Fluorescence images were taken with a Zeiss LSM510 confocal microscope (White Plains, NY).

### Cytokine array and ELISA

Fresh fistula tissue was homogenized, and protein was extracted with radioimmunoprecipitation assay buffer. A BCA kit was used to measure protein concentrations. Proteome Profiler Rat Cytokine Array Kit, Panel A (ARY008, R&D Systems, Minneapolis, MN) was used to determine the cytokine profile in different fistula treatment groups. Four hundred micrograms of protein was used for the assay following the manufacture’s instruction. IRDye@800CM Streptavidin was used to detect the protein signal. Array data were collected with LI-COR Odyssey INFRARED Imaging System (LI-COR Biosciences, Lincoln, NE) and analyzed with ImageJ Fiji (NIH, Bethesda, MD). Target cytokines TNF-α, IL-6, and IL-10 were selected for analysis in all treatment groups. ELISA assay was performed following the protocol from ELISA kits as shown below. Data were collected on a microplate reader (BioTek Elx × 808, Winooski, VT).

### Flow cytometry

mfNHC and fistula tissue were collected from rats, minced into small pieces, and digested with collagenase (2.5 mg/ml; C5138, Sigma-Aldrich, Burlington, MA) or hyaluronidase (7 mg/ml; H3506, Sigma-Aldrich, Burlington, MA) at 37°C for 0.5 to 1 hour. The cell suspension was filtered with 70-μm strainers, and single cells were collected. 7-Aminoactinomycin D (Thermo Fisher Scientific, Waltham, MA) was used to stain for dead cells, and cells were blocked with Fc blocker. Cell membrane protein staining was carried following the standard cell membrane flow cytometry protocol (BD Biosciences, San Diego, CA). Next, cells were fixed and permeabilized. Intracellular protein CD68 was stained with the standard intracellular protein protocols as described (*49*, *50*). Data were collected with the SONY SH800 Cell sorter machine (Sony Biotechnology, San Jose, CA). The flow cytometry data were analyzed using FlowJo v10.6 software (BD Biosciences, Franklin Lakes, NJ).

### Antibodies

The following reagents were used in the studies: anti-CD68 mouse antibody (ab31630, Abcam, Cambridge, UK), anti-CD68 rabbit antibody (ab125212, Abcam, Cambridge, UK), Alexa Flour 647 anti-rat CD68/SR-D1 (Novus Biologicals, Littleton, CO), anti-CD86 mouse antibody (ab213044, Abcam, Cambridge, UK), anti-CD38 antibody (GTX37752, GeneTex, Irvine, CA), recombinant anti-CD163 rabbit antibody (ab182422, Abcam, Cambridge, UK), phycoerthrin (PE)/Cy5.5 anti-rat CD163 (Novus Biologicals, Littleton, CO), PE anti-rat CD38 (BioLegend, San Diego, CA), anti–α-SMA rabbit antibody (ab5694, Abcam, Cambridge, UK), mouse CD45RO antibody (MA5-11532, Thermo Fisher Scientific, Waltham, MA), anti-CD20 antibody (70168, Cell Signaling Technology, Danvers, MA), Cytokeratin Pan Type I/II Antibody Cocktail mouse (MA1-82041, Thermo Fisher Scientific, Waltham, MA), Alexa Fluor 594 or 488 and 647 secondary antibodies (Life Technologies, Carlsbad, CA), Rat BD Fc Block (550270, BD Biosciences, San Jose, CA), BV421 mouse anti-rat CD3 (563948, BD Biosciences, San Jose, CA), peridin chlorophyll protein (PerCP)/Cyanine5.5 anti-rat CD11b/c antibody (201819, BioLegend, San Diego, CA), PE/Cyanine7 anti-rat CD45 antibody (202213, BioLegend, San Diego, CA), CD68/SR-D1 antibody (ED1) [Alexa Fluor 405] (NB600-985/AF405, Novus Biologicals, Littleton, CO), PE mouse anti-rat CD86 (551396, BD Biosciences, San Jose, CA), PE/Cyanine7 anti-rat CD161 antibody (205609, BioLegend, San Diego, CA), PE/Cyanine7 anti-rat CD161 antibody (202307, BioLegend, San Diego, CA), fluorescein isothiocyanate (FITC) anti-mouse/rat CD29 antibody (102205, BioLegend, San Diego, CA), PE mouse anti-rat CD4 (551397, BD Biosciences, San Jose, CA), allophycocyanin (APC) mouse anti-rat CD90/mouse CD90.1 (561409, BD Biosciences, San Jose, CA), PE anti-rat CD106 antibody (200403, BioLegend, San Diego, CA), CD31 monoclonal antibody (TLD-3A12), FITC (MA5-16952, Thermo Fisher Scientific, Waltham, MA), FITC anti-rat CD45 antibody (202205, BioLegend, San Diego, CA), rat IL-6 DuoSet ELISA kit (DY522-05, R&D Systems, Minneapolis, MN), rat IL-10 DuoSet ELISA kit (DY506-05, R&D Systems, Minneapolis, MN), and rat TNFα ELISA kit (ab100785, Abcam, Cambridge, UK).

### Statistics

Graphs were generated with GraphPad Prism 9 (GraphPad, San Diego, CA) and TB tool (*51*). Data were shown as means ± SD or means ± SEM, with details shown in figure legends. Statistical significance was determined with a two-tailed Student’s *t* test or one-way analysis of variance. A two-sided *P* values of <0.05 (*) or 0.01 (**) or 0.001 (***) were considered statistically significant.

## Notes

View/request a protocol for this paper from *Bio-protocol*.
