# Supplementary material for: A nanofiber-hydrogel composite improves tissue repair in a rat model of Crohn’s disease perianal fistulas
Source: Sci Adv. 2023 Jan 4;9(1):eade1067. doi: 10.1126/sciadv.ade1067 (PMC9812382; doi:10.1126/sciadv.ade1067)
Supplement: Supplementary file 1 — Table S1 Figs. S1 to S16 [file sciadv.ade1067_sm.pdf]

Science Advances
AAAS

Supplementary Materials for

A nanofiber-hydrogel composite improves tissue repair in a rat model of Crohn's disease perianal fistulas

Ling Li *et al.*

Corresponding author: Hai-Quan Mao, hmao@jhu.edu; Florin M. Selaru, fselaru1@jhmi.edu

Sci. Adv. **9**, eade1067 (2022)
DOI: 10.1126/sciadv.ade1067

The PDF file includes:

Table S1
Figs. S1 to S16
Legends for movies S1 and S2

Other Supplementary Material for this manuscript includes the following:

Movies S1 and S2

Supplementary Table 1. Rat fistula groups for treatment

Rat ID	Right fistula	Left fistula
Rat 1	mfNHC-250	Surgery alone
Rat 2	mfNHC-250	Surgery alone
Rat 3	mfNHC-250	Surgery alone
Rat 4	ADSC-mfNHC-250	Surgery alone
Rat 5	ADSC-mfNHC-250	Surgery alone
Rat 6	ADSC-mfNHC-250	Surgery alone
Rat 7	ADSC-mfNHC-250	mfNHC-250
Rat 8	ADSC-mfNHC-250	mfNHC-250
Rat 9	ADSC-mfNHC-250	mfNHC-250

Supplementary Figures:

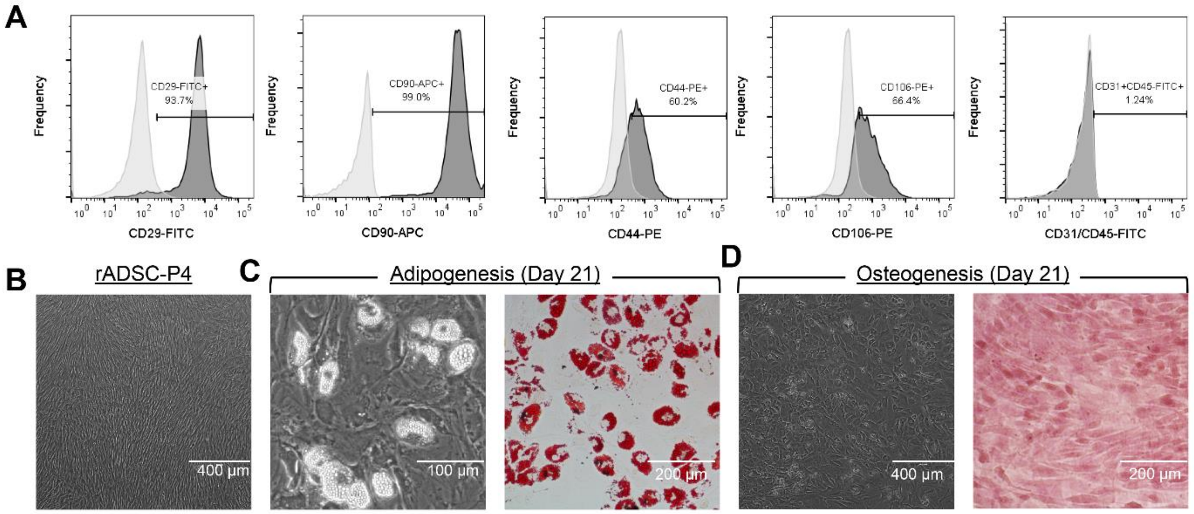

Supplementary Figure 1. Isolation, characterization and differentiation of rat ADSCs. (A) Flow cytometry analysis showing that ADSCs are positive for MSC markers CD29, CD90, CD44 and CD106, but negative for the hematopoietic cell marker CD45 and the endothelial cell marker CD31. (B) Bright light image showing typical spindle-shaped morphology of primary rat ADSC at passage 4 (scale bar: 400 µm). (C) Oil Red-O staining of lipids in ADSCs differentiated towards adipocytes (left panel scale bar: 100 µm, right panel scale bar: 200 µm). (D) Alizarin Red staining showing staining of calcium containing osteocytes in ADSC differentiated towards osteocytes. (left panel scale bar: 400 µm, right panel scale bar: 200 µm).

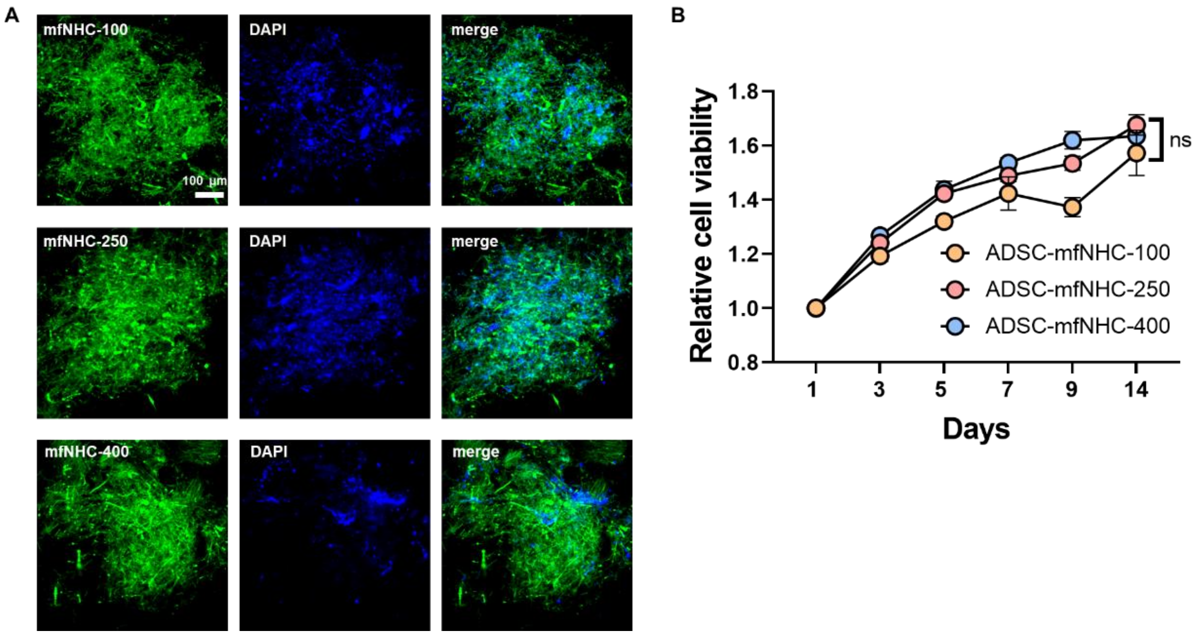

Supplementary Figure 2. *In vitro* growth characteristics of ADSCs on mfNHC. (A) Confocal image of ADSCs grown on and into mfNHC at 3 different levels of stiffness. (Green: F8BT-labeled nanofibers, Blue: DAPI-labeled nuclei). (B) AlamarBlue cell proliferation assay showed the ADSC proliferation on mfNHC for a period of 14 days (n = 6; ns: no significance).

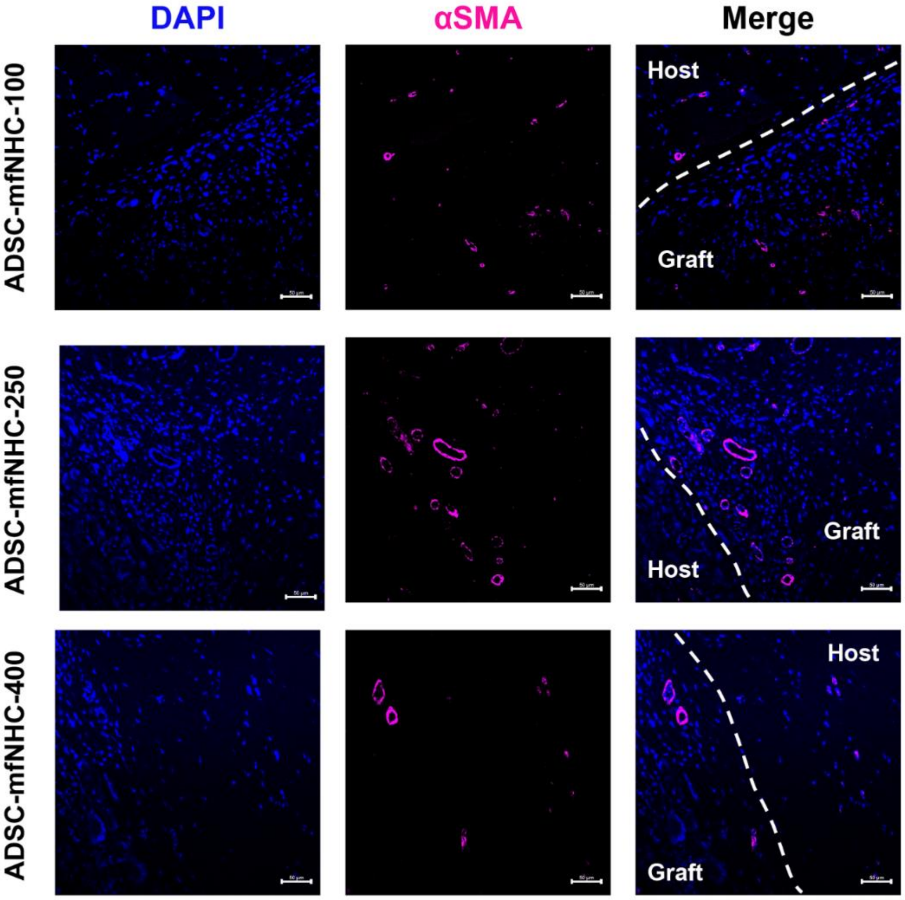

Supplementary Figure 3. Immunofluorescence (IF) of mfNHC samples of 3 different levels of storage modulus. IF staining showing new micro-vessel formation at day 14 days after the implantation of ADSC-mfNHC via S.C injection. Pink - αSMA, Blue - DAPI (scale bar: 50 μm).

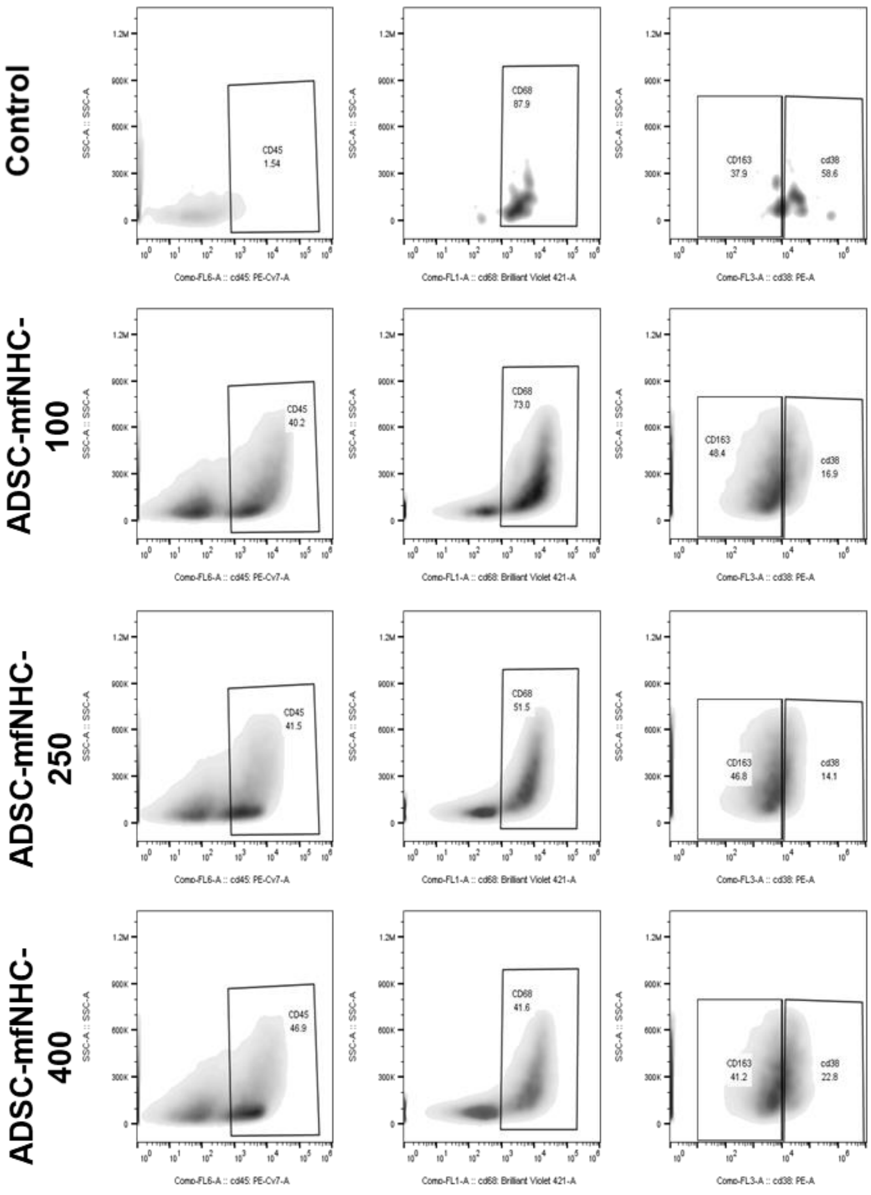

Supplementary Figure 4. Flow cytometry density plot of macrophage populations at day 3 in s.c. implants of ADSC-mfNHC of 3 different levels of storage modulus.

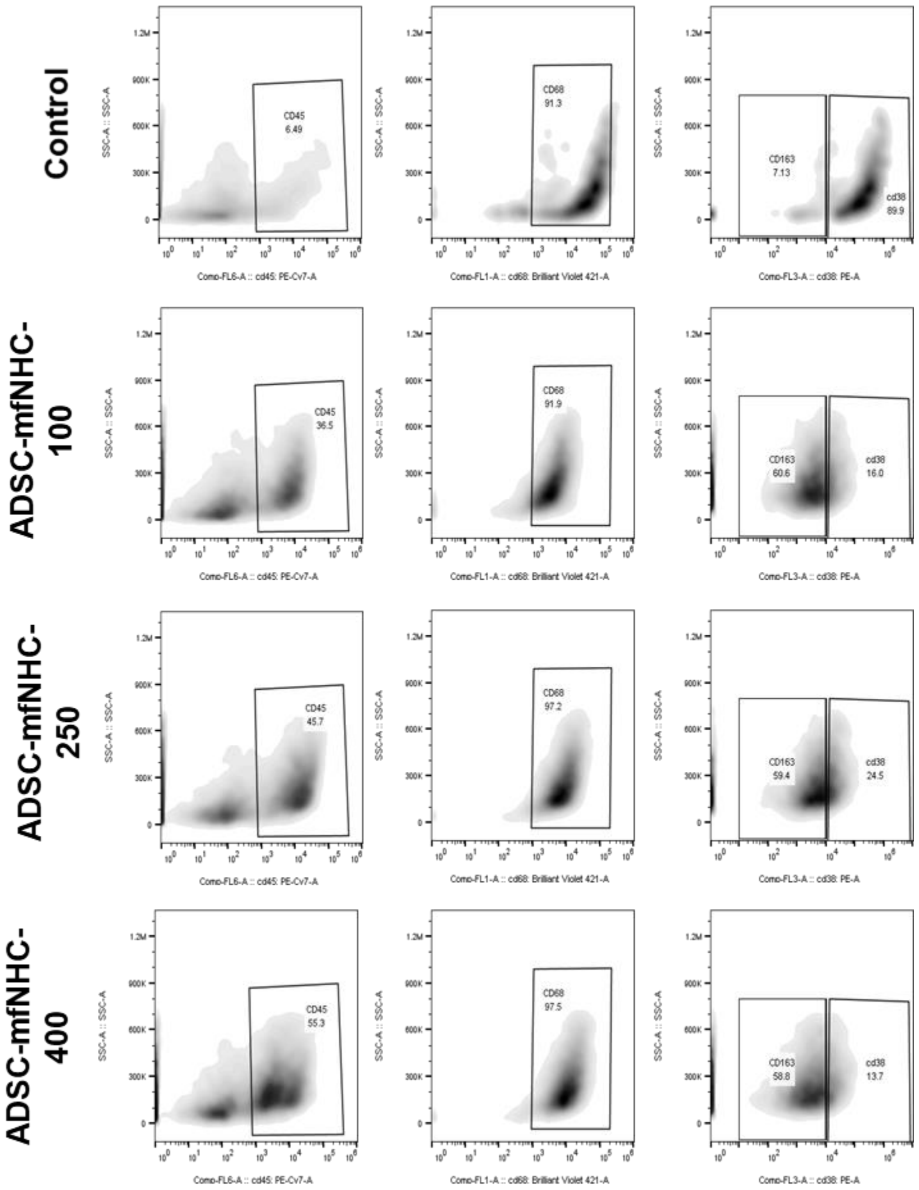

Supplementary Figure 5. Flow cytometry density plot of macrophage populations at day 14 in s.c. implants of ADSC-mfNHC of 3 different levels of storage modulus.

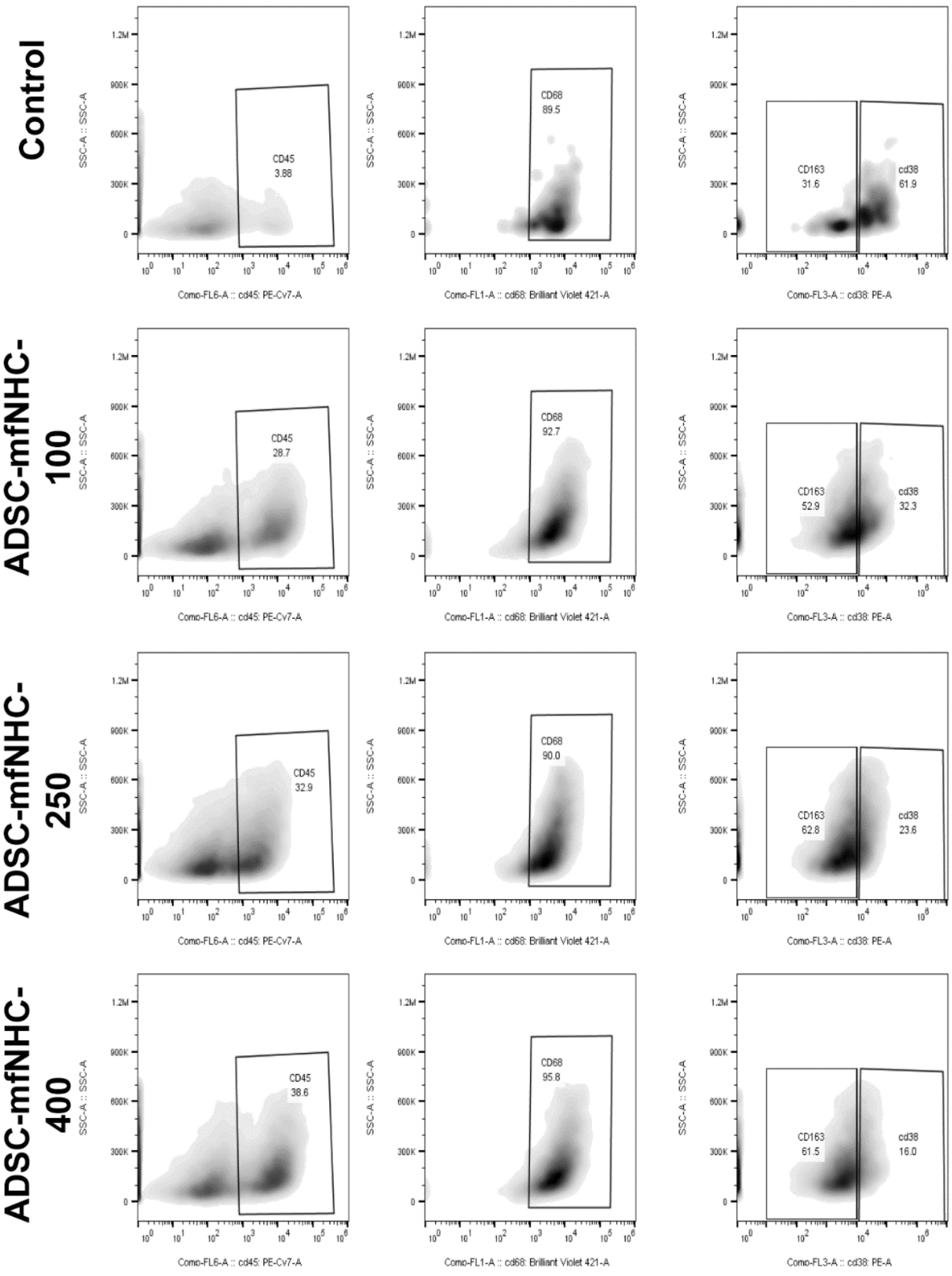

Supplementary Figure 6. Flow cytometry density plot of macrophage populations at day 28 in s.c. implants of ADSC-mfNHC of 3 different levels of storage modulus.

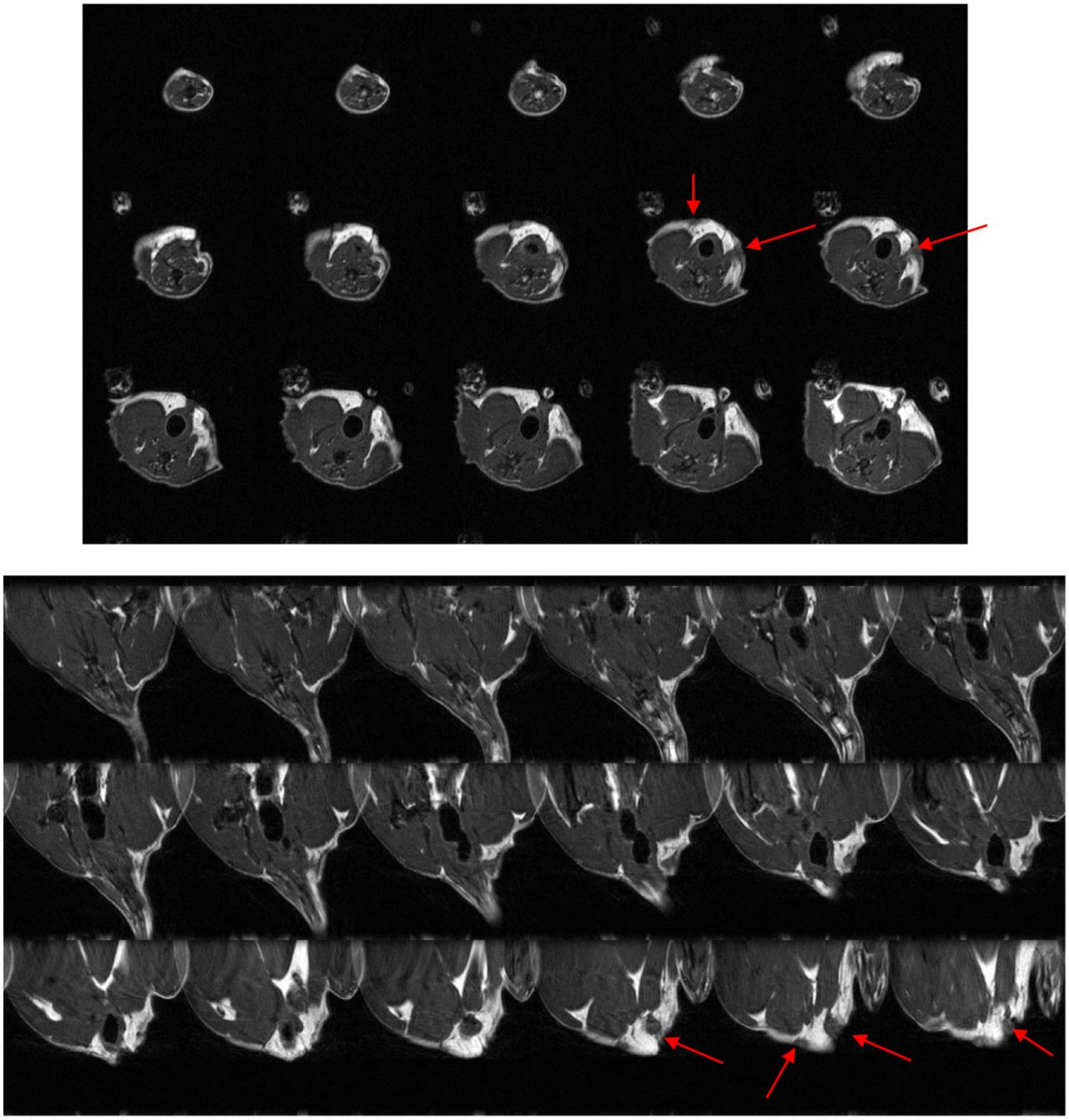

Supplementary Figure 7. Axial and coronal T1 MRI images for treated fistula of rat No. 1. MRI images show moderate healing of the right sided fistula (right: mfNHC-250), and persistent left fistula (left: surgery alone), The maximal length and width of surgery alone treatment fistula are 4.59 mm and 5.48 mm, while the maximal length and width of mfNHC-250 treatment fistula are 1.80 mm and 3.11 mm.

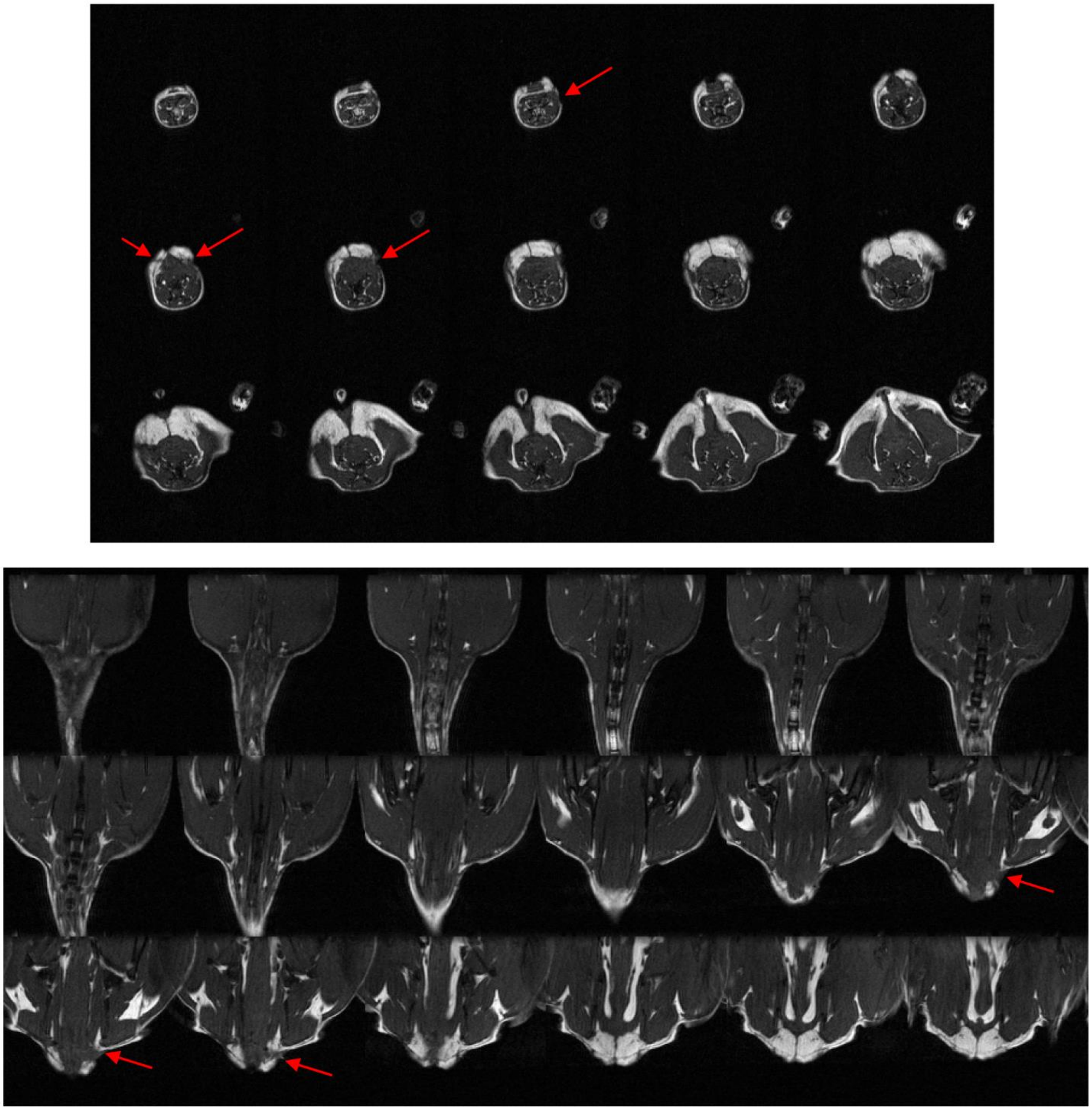

Supplementary Figure 8. Axial and coronal T1 MRI images for treated fistula of rat No. 2. MRI images show mild to moderate healing of the right sided fistula (right: mfNHC-250), and persistent left fistula (left: surgery alone). the maximal length and width of surgery alone treatment fistula are 2.04 mm and 4.93 mm, while the maximal length and width of mfNHC-250 treatment fistula are 3.68 mm and 2.25 mm.

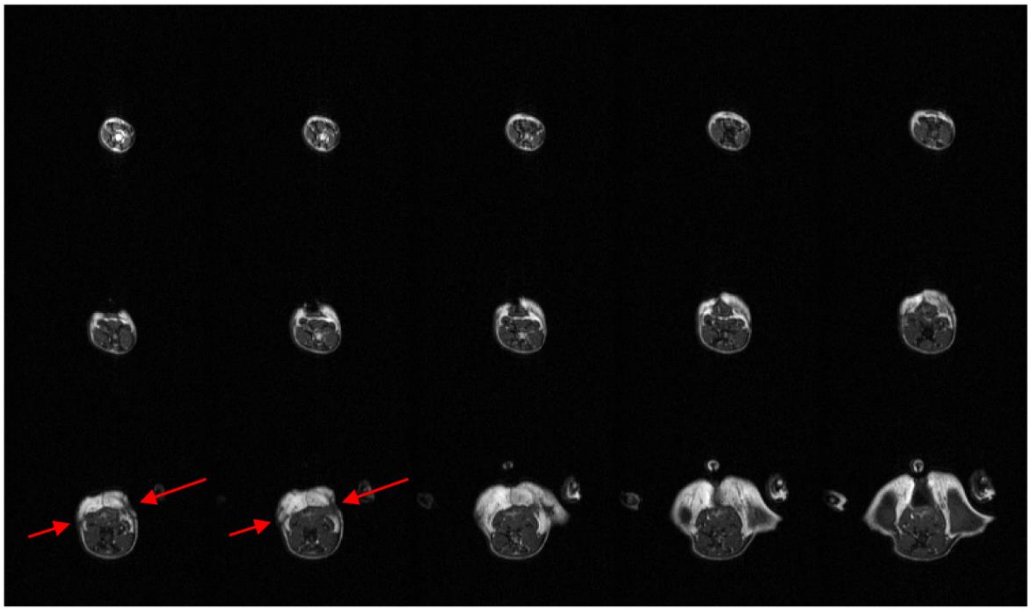

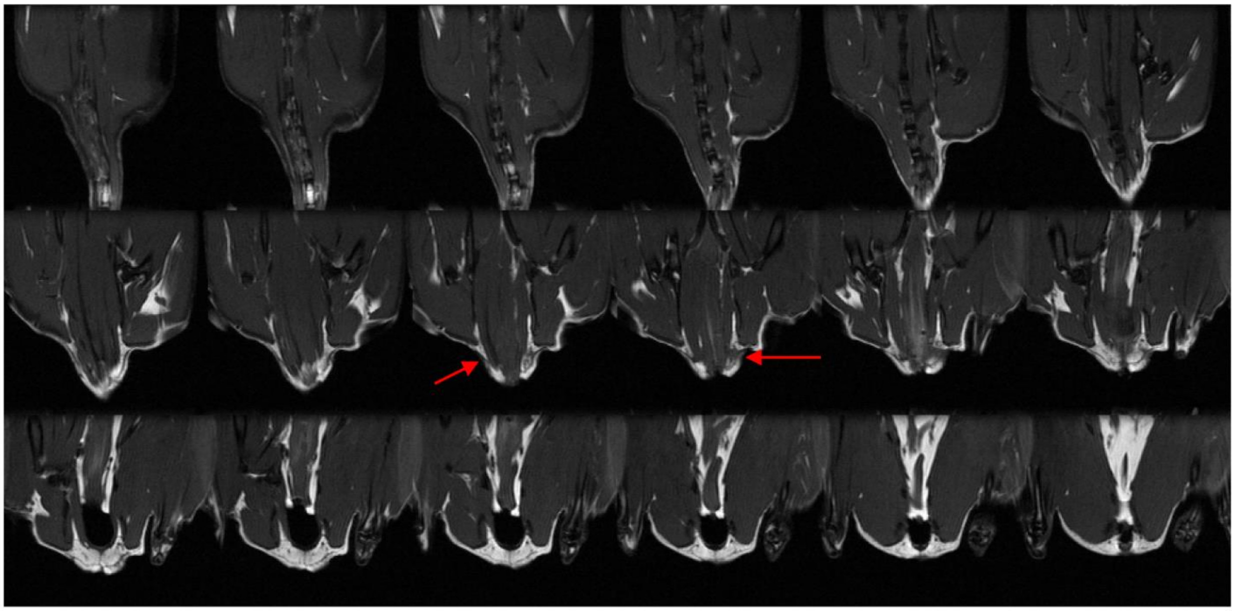

Supplementary Figure 9. Axial and coronal T1 MRI images for treated fistula of rat No. 3. MRI images show moderate to complete healing of the right sided fistula (right – mfNHC-250), and persistent left fistula with mild healing (left – surgery alone). The maximal length and width of surgery alone treatment fistula are 4.61 mm and 3.12 mm, while the maximal length and width of mfNHC-250 treatment fistula are 1.65 mm and 2.03 mm.

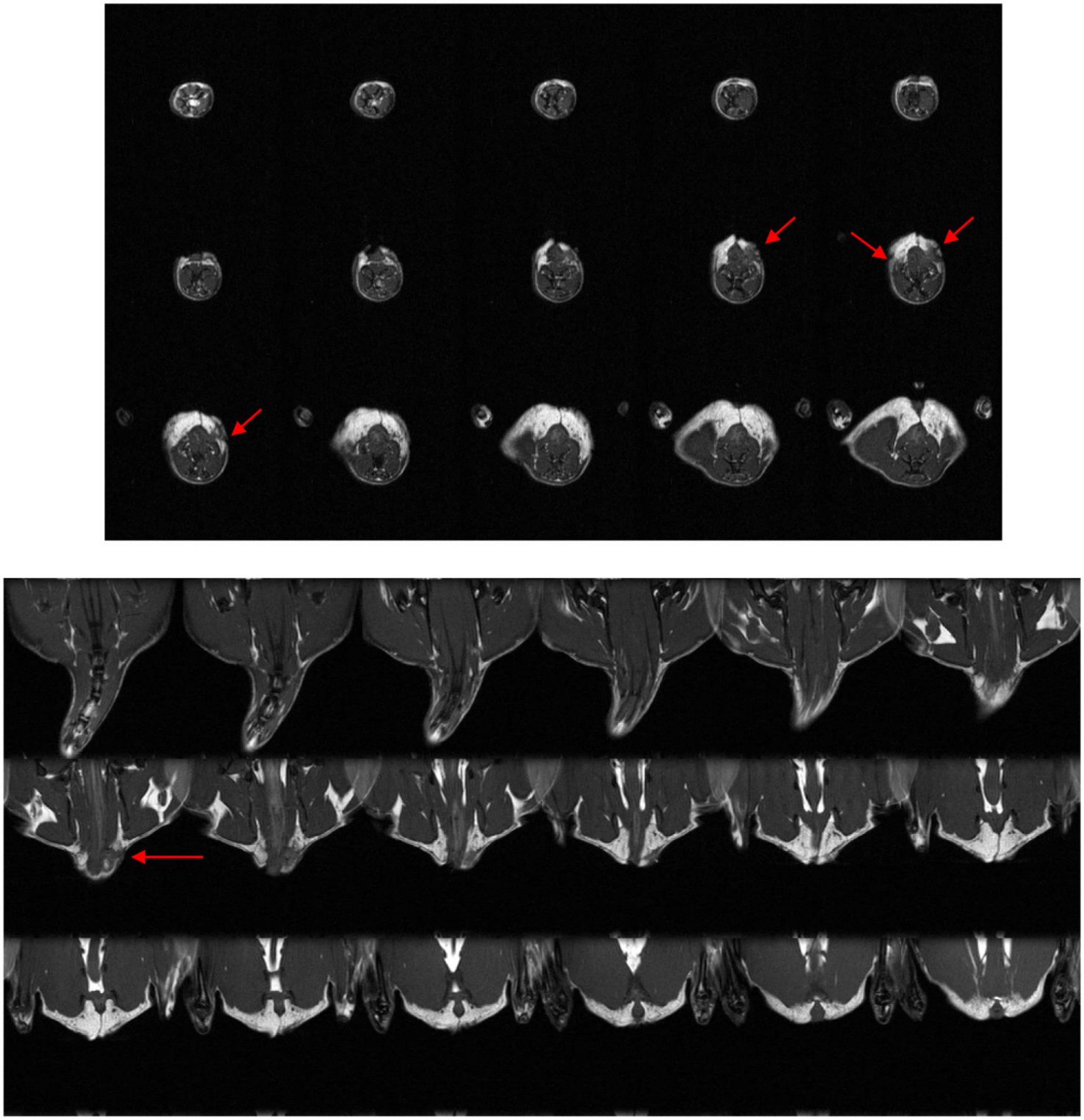

Supplementary Figure 10. Axial and coronal T1 MRI images for treated fistula of rat No. 4. MRI images show nearly complete healing of the right sided anal fistula (right –ADSC-mfNHC-250), and persistent left fistula (left – surgery alone). The maximal length and width of surgery alone treatment fistula are 3.40 mm and 4.63 mm, while the maximal length and width of ADSC-mfNHC-250 treatment fistula are 1.01 mm and 1.35 mm.

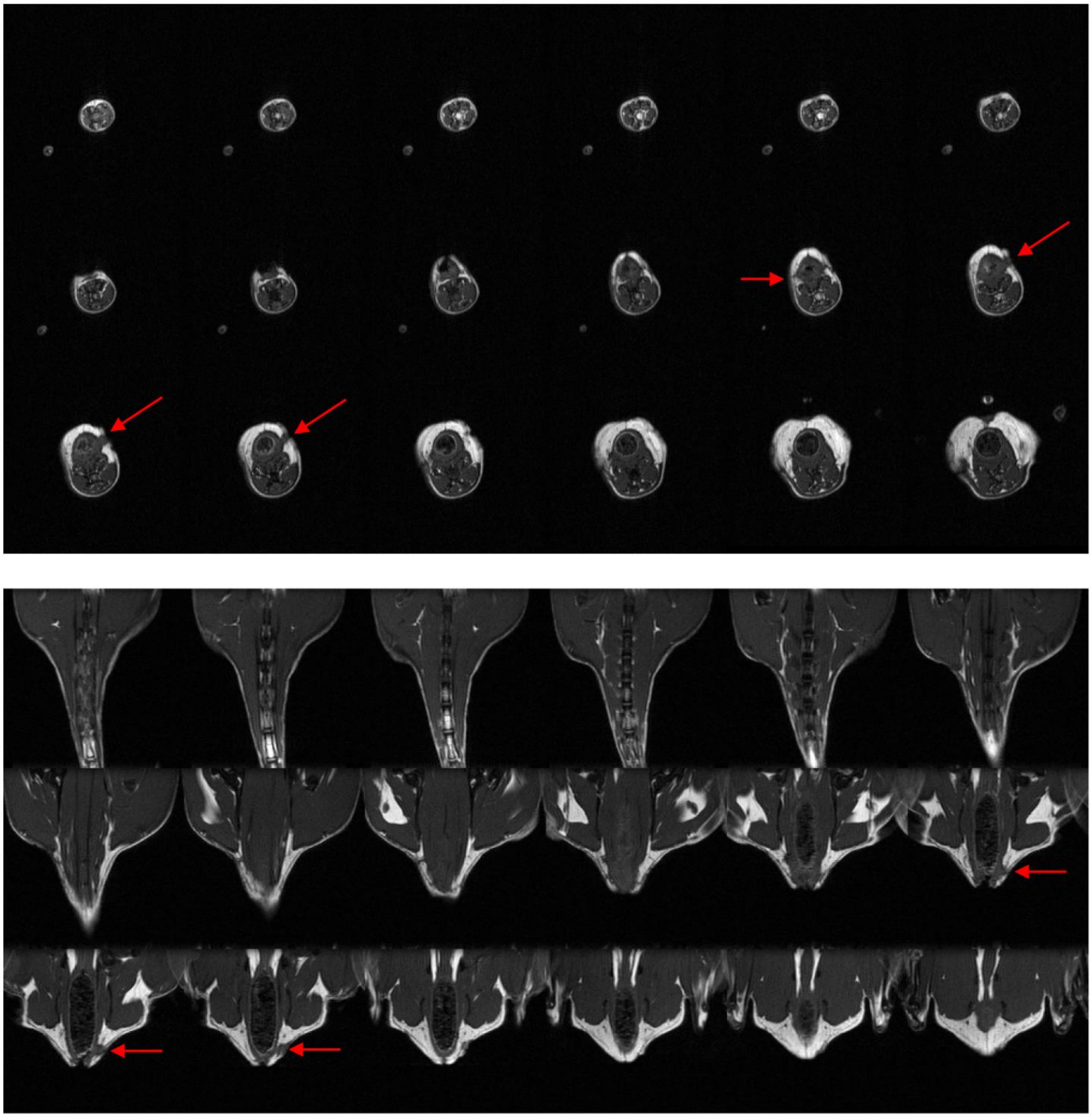

Supplementary Figure 11. Axial and coronal T1 MRI images for treated fistula of rat No. 5. MRI images show moderate to near complete healing of the right sided fistula (right – ADSC-mfNHC-250), and mild to moderate healing of left fistula (left – surgery alone). The maximal length and width of surgery alone treatment fistula are 2.62 mm and 2.83 mm, while the maximal length and width of ADSC-mfNHC-250treatment fistula are 1.16 mm and 2.54 mm.

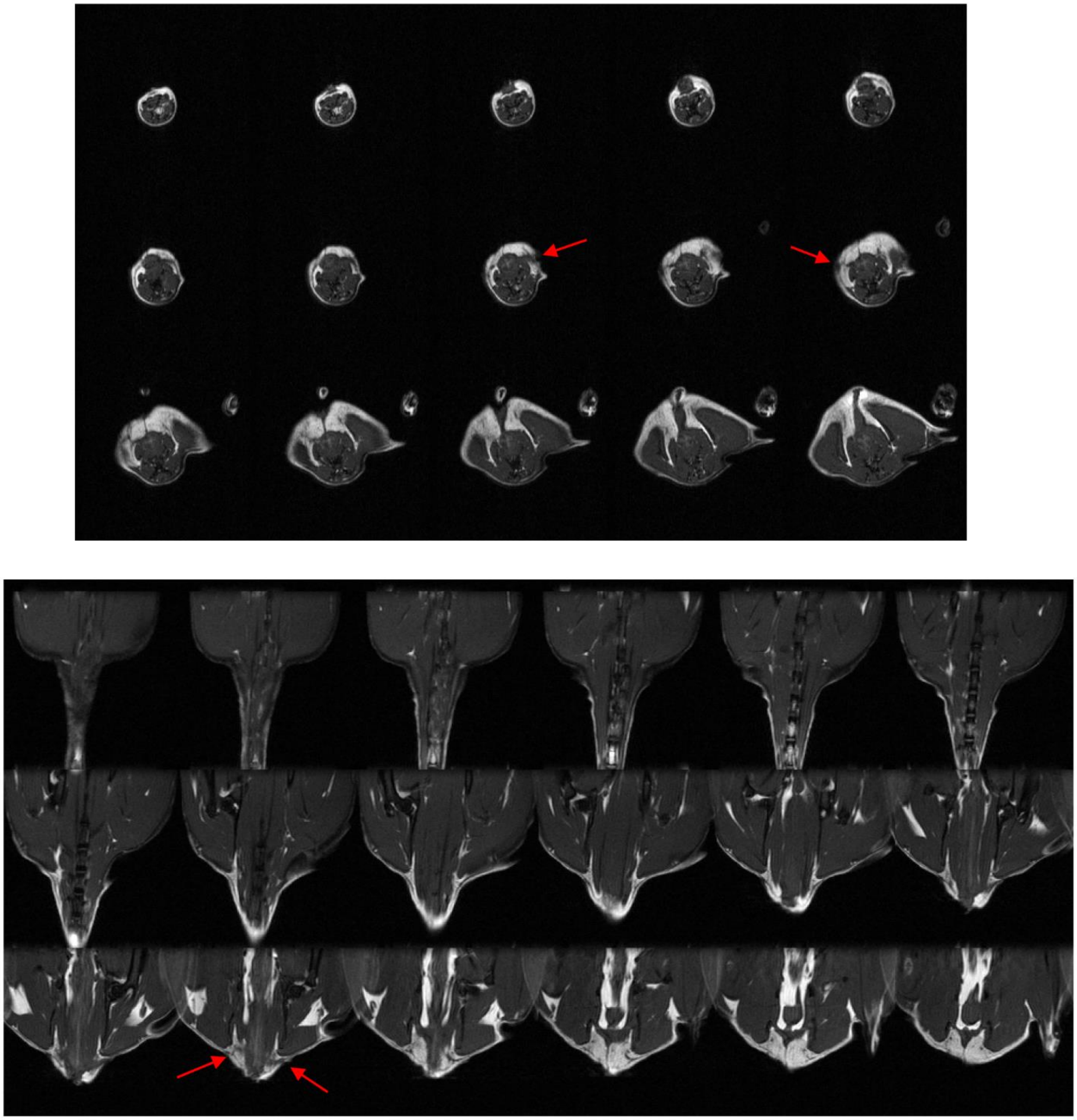

Supplementary Figure 12. Axial and coronal T1 MRI images for treated fistula of rat No. 6. MRI images show nearly complete healing of the right sided fistula (right – ADSC-mfNHC-250), and persistent open with slight healing of left fistula (left – surgery alone). The maximal length and width of surgery alone treatment fistula are 3.00 mm and 3.23 mm, while the maximal length and width of ADSC-mfNHC-250treatment fistula are 1.33 mm and 1.90 mm.

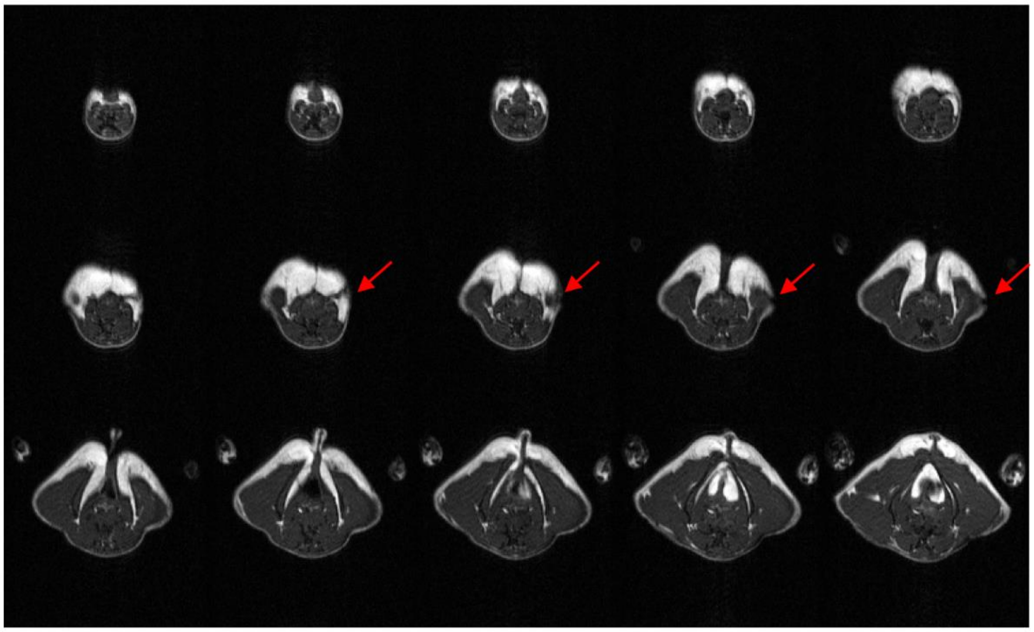

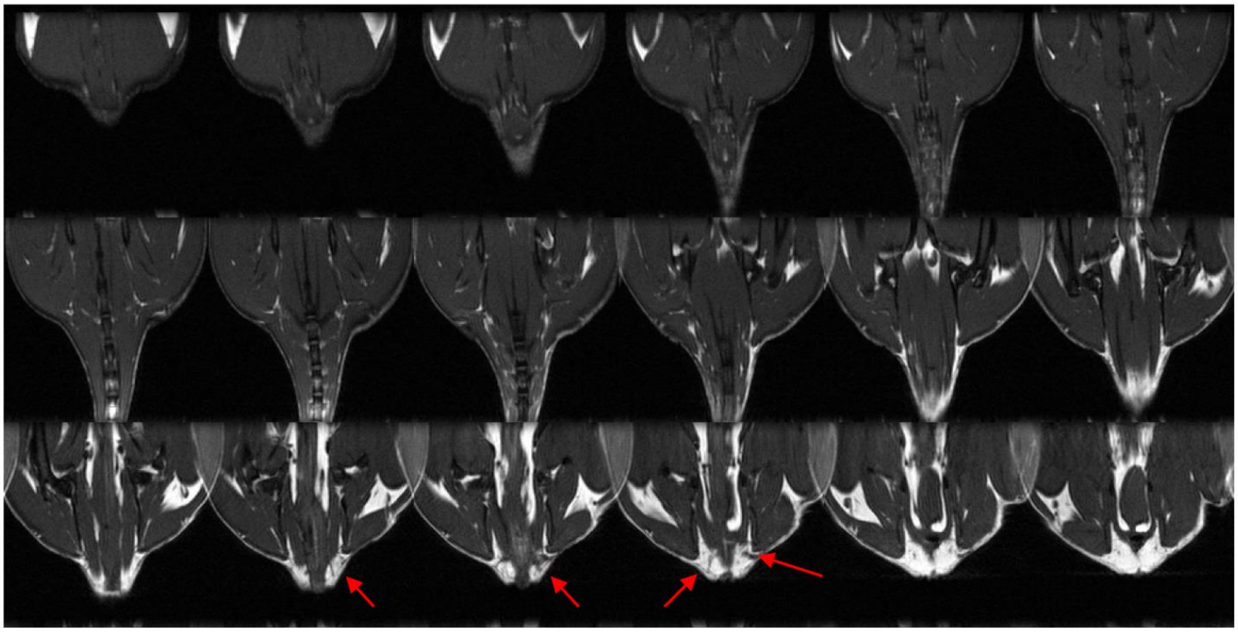

Supplementary Figure 13. Axial and coronal T1 MRI images for treated fistula of rat No. 7. MRI images show complete healing of the right sided fistula (right – ADSC-mfNHC-250), and persistent left fistula (left – mfNHC-250). The maximal length and width of mfNHC-250 treatment fistula are 5.15 mm and 3.30 mm, while the maximal length and width of ADSC-mfNHC-250 treatment fistula are 0 mm and 0 mm.

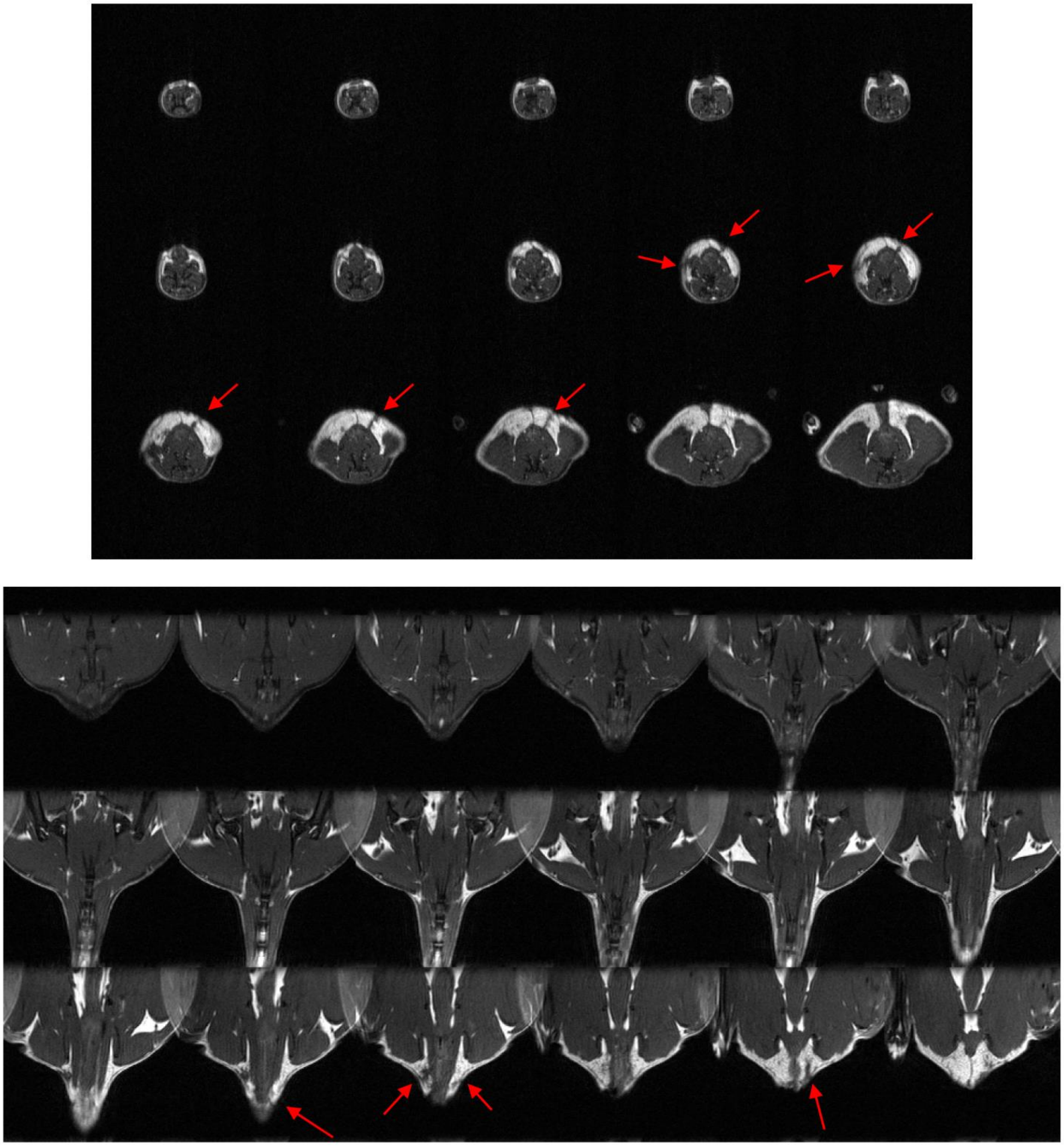

Supplementary Figure 14. Axial and coronal T1 MRI images for treated fistula of rat No. 8. MRI images show similar mild to moderate healing of both sides of fistulas (right – ADSC-mfNHC-250), (left – mfNHC-250). The maximal length and width of mfNHC-250 treatment fistula are 5.17 mm and 1.50 mm, while the maximal length and width of ADSC-mfNHC-250treatment fistula are 3 mm and 3.0 mm.

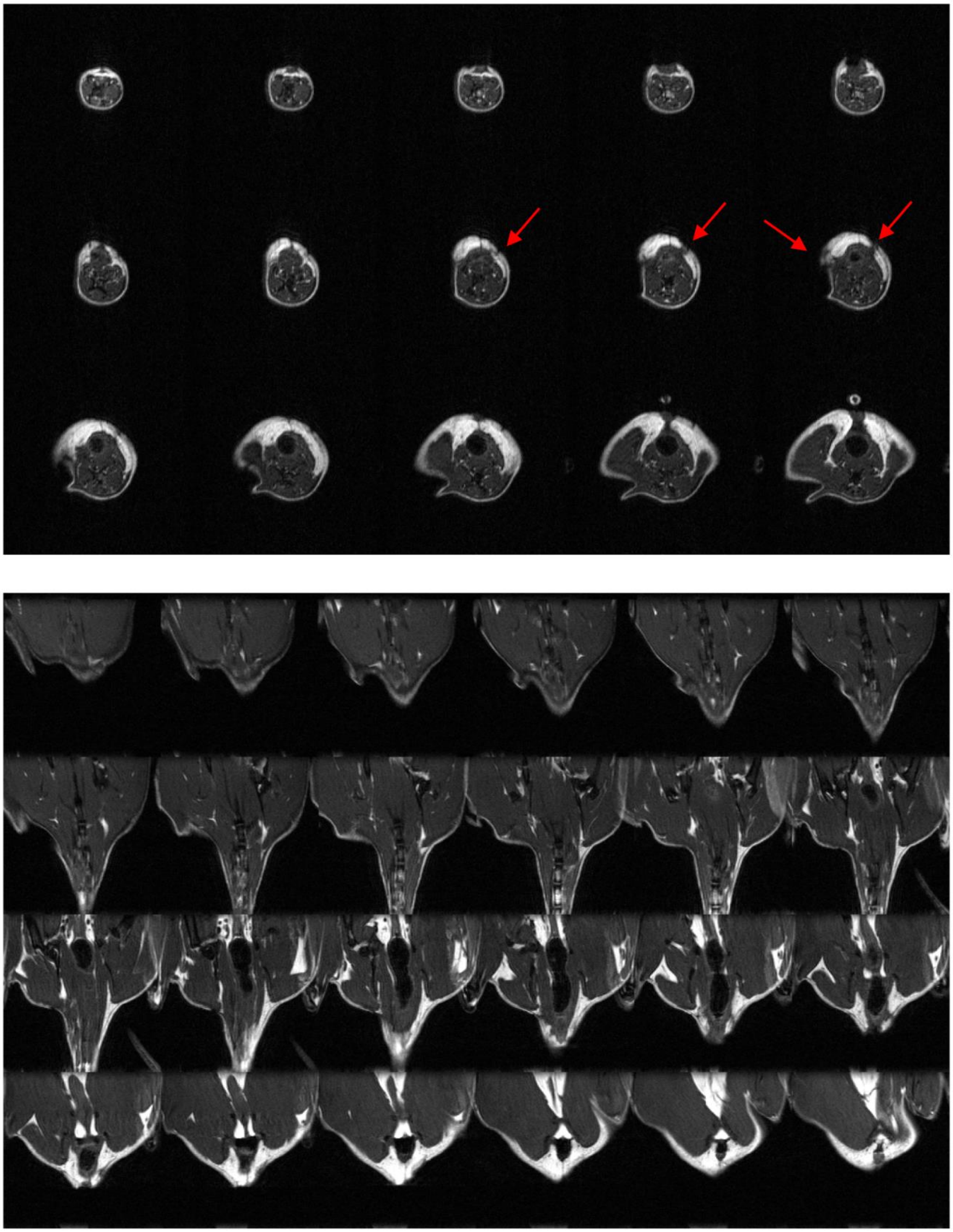

Supplementary Figure 15. Axial and coronal T1 MRI images for treated fistula of rat No. 9. MRI images show nearly similar moderate to complete healing of both fistulas. The right side healed better than left side. (right – ADSC-mfNHC-250), (left – mfNHC-250). The maximal length and width of mfNHC-250 treatment fistula are 2.24 mm and 2.00 mm, while the maximal length and width of ADSC-mfNHC-250treatment fistula are 1.17 mm and 1.80 mm.

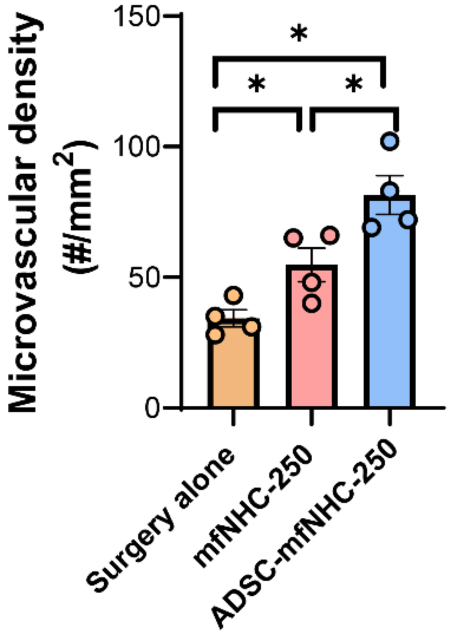

Supplementary Figure 16. Quantification of microvascular density in Figure 4C. The results showed ADSC-mfNHC-250 treatment group has the largest amount of microvascularity compared with the mfNHC-250 as well as surgery alone; (n = 6, $*P < 0.05$).

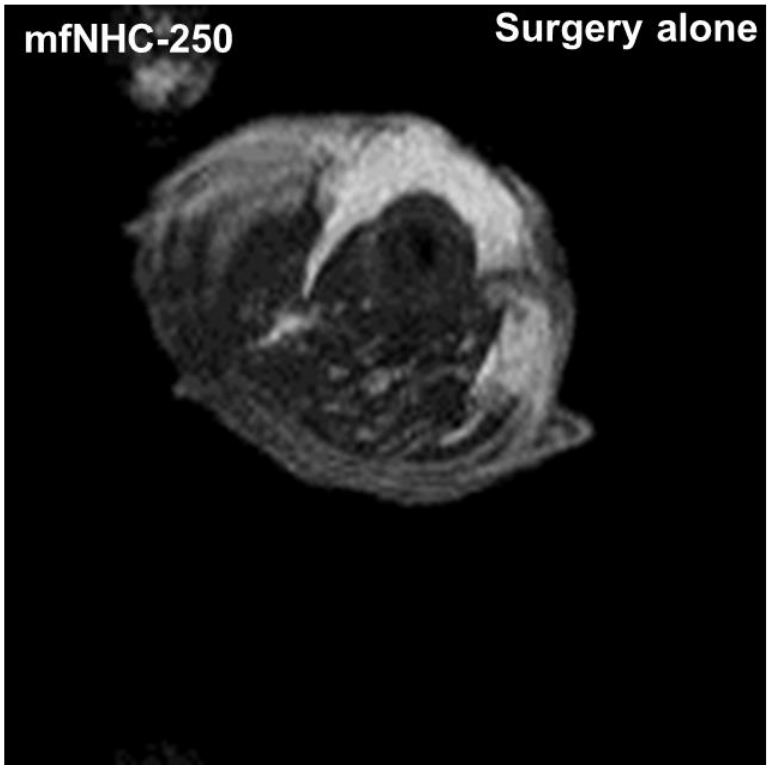

Supplementary Video 1: MRI 3D video of Rat 1 that treated with mfNHC-250 (right), and surgery alone (left).

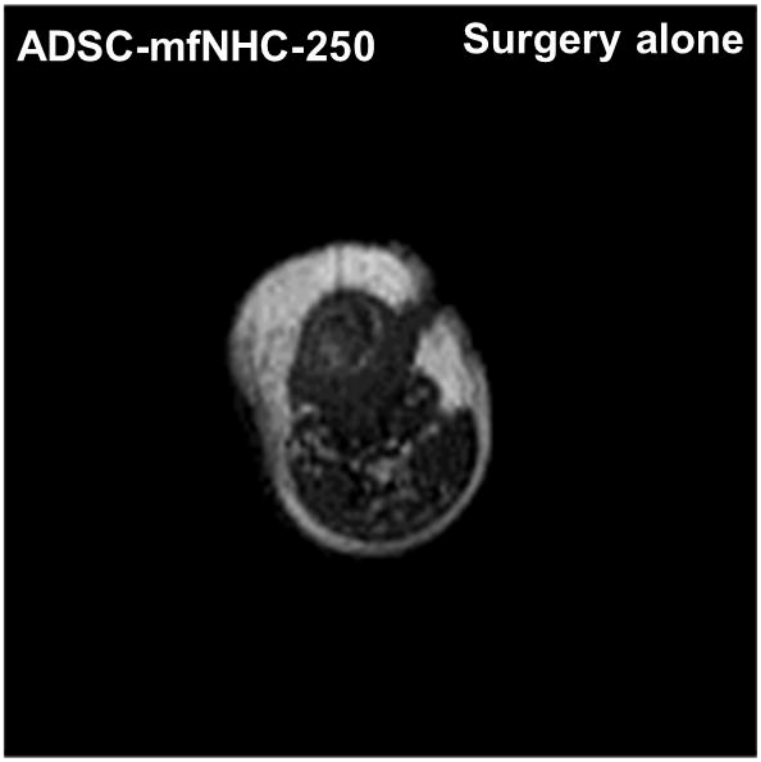

Supplementary Video 2: MRI 3D video of Rat 5 that treated with ADSC-mfNHC-250 (right), and surgery alone (left).
